# Manipulating the fluorescence lifetime at the sub-cellular scale via photo-switchable barcoding

**DOI:** 10.1038/s41467-020-16297-3

**Published:** 2020-05-18

**Authors:** Yujie Xie, Maria C. Arno, Jonathan T. Husband, Miquel Torrent-Sucarrat, Rachel K. O’Reilly

**Affiliations:** 1 School of Chemistry, University of Birmingham, Edgbaston, Birmingham, B15 2TT UK; 2 Department of Chemistry, University of Warwick, Coventry, CV4 7AL UK; 3 Department of Organic Chemistry I, Universidad del País Vasco (UPV/EHU), and Donostia International Physics Center (DIPC), Manuel Lardizabal Ibilbidea 3, Donostia, 20018 Spain; 4 Ikerbasque, Basque Foundation for Science, María Díaz de Haro 3, Bilbao, 48013 Spain

**Keywords:** Polymers, Supramolecular polymers, Polymers

## Abstract

Fluorescent barcoding is a pivotal technique for the investigation of the microscale world, from information storage to the monitoring of dynamic biochemical processes. Using fluorescence lifetime as the readout modality offers more reproducible and quantitative outputs compared to conventional fluorescent barcoding, being independent of sample concentration and measurement methods. However, the use of fluorescence lifetime in this area has been limited by the lack of strategies that provide spatiotemporal manipulation of the coding process. In this study, we design a two-component photo-switchable nanogel that exhibits variable fluorescence lifetime upon photoisomerization-induced energy transfer processes through light irradiation. This remotely manipulated fluorescence lifetime property could be visually mapped using fluorescence lifetime imaging microscopy (FLIM), allowing selective storage and display of information at the microscale. Most importantly, the reversibility of this system further provides a strategy for minimizing the background influence in fluorescence lifetime imaging of live cells and sub-cellular organelles.

## Abstract (web summary)

Using fluorescence lifetime as the readout modality offers more reproducible and quantitative outputs compared to conventional fluorescent barcoding, being independent of sample concentration and measurement methods. Here, the authors design a photo-switchable nanogel exhibiting variable fluorescence lifetime, and demonstrate visual mapping by using fluorescence lifetime imaging microscopy on a sub-cellular scale.

## Introduction

With increasing attention directed to exploring microscale events, more effective tools are needed to fully understand processes at the micro-level^1^. Micro-barcoding is a versatile technique that provides multiplex and high-throughput information storage for micro- and nanoscale applications across the fields of biological, medicinal, and material sciences^2,3^. Particularly, optical multiplexing or fluorescent barcoding has recently attracted increasing interest, largely owing to its high sensitivity, fast signaling, and minimally invasive non-destructive nature^4–8^. However, current fluorescent barcoding techniques mainly rely on the use of spectral multiplexing and fluorescence intensity (FI) encoding, which are typically susceptible to spectral overlap of the encoding elements. Moreover, obtaining a quantitative readout is a major challenge as a consequence of the variability of sample concentration and external microenvironment.

One of the forefront techniques to provide reproducible output in the micro barcoding area is utilizing fluorescence lifetime, an intrinsic photophysical property that is independent of the local fluorophore concentration and the technique used for measurement^9,10^. With state-of-the-art microscopic imaging techniques, fluorescence lifetime could be used as a straightforward technology to minimize the limitations in traditional fluorescent barcoding, providing a reproducible and quantitative readout over time^10–12^. Current research into lifetime barcoding has predominantly exploited inorganic fluorescent materials containing lanthanides and transition metals ions, in which the fluorescence lifetime can be modulated by altering their structural configuration and composition during synthesis^13–16^. Alternatively, fluorescence lifetime can be tuned by modifying the efficiency of energy transfer between different molecules^17,18^. Hildebrandt and co-workers have revealed detailed insight into the energy transfer between lanthanide complexes and semiconductor quantum dots, leading to a photoluminescence lifetime platform for a variety of applications including bioanalysing, imaging, and information storage^19–22^. Despite these advances, the search for responsive materials in which the fluorescence lifetime can be flexible and adjustable in real-time is still an ongoing challenge^23^. Alternatively, polymeric nanoparticles can offer great advantages in this area, owing to their ease of functionalization and capability to quickly respond to external stimuli^24^.

Substituted maleimides, a range of versatile small molecule fluorophores, are distinguished by their small size and bright fluorescence emission, but most importantly their fluorescent properties can be tuned by carefully modifying the substituents and are dependent upon the fluorophore microenvironment^25–27^. As previously reported by our group, the encapsulation of dithiomaleimides (DTM) in a hydrophobic environment, such as the core of an amphiphilic polymeric assembly, significantly increases the fluorescence lifetime by eliminating both self-quenching caused by DTM aggregation and collisional quenching from the surrounding solvent^28,29^. In comparison to inorganic fluorophores, small molecule fluorophores like substituted maleimides are promising as a consequence of lower toxicity and easily tuneable fluorescent properties, therefore representing a potential tool for barcoding applications^30–33^.

Light, as a non-invasive stimulus, is one of the prominent tools used to achieve external manipulation without the need for physical contact, hence being ideal for information imprinting, real-time labeling, and selective tracking^34–38^. In this study, we design a light-switchable lifetime barcoding system based on two-component nanogels involving substituted maleimides and a spiropyran (SP) switch. By inducing reversible photoisomerization processes, nanogels with similar structures but multiplex lifetimes could be realized via Förster Resonance Energy Transfer (FRET) between the DTM fluorophore to the ring-opened form of the SP photochrome, allowing for dynamic fluorescence lifetime coding in a controllable, non-invasive fashion. Moreover, the multi-states of these light-switchable lifetime barcodes benefit from the self-correction technique which could be used to increase the sensitivity of the fluorescence lifetime imaging microscopy and provide a high level of information stored at the micro-scale. As a proof-of-concept, the switchable nanogel is functionalized with mitochondria targeting group and visualize at both the cellular and subcellular scales in living cells, showing the applicability of this system in monitoring the cellular microenvironment.

## Results

### Design and preparation of photo-switchable nanogels

Fluorescence lifetime, as a direct measurement of the time-resolved fluorescence decay, is closely related to the structure of the molecule and the interaction with its microenvironment^39^. In this study, the tailoring of fluorescence lifetime relies on modulating the FRET, which has been proved to be a practical tool for controlling fluorescence properties through long-range dipole–dipole coupling^18^. DTM was chosen as a fluorescence donor owing to its strong emission and sufficiently long fluorescence lifetime in a hydrophobic environment^28,29,40^. Compared with commonly used small-molecule fluorophores, the uniquely high fluorescence lifetime of DTM dyes in hydrophobic environments allows both (i) a relatively wide lifetime range for more diverse fluorescence lifetimes with minimum overlap and (ii) high resolution in a biologically relevant context, representing a distinct advantage over commonly used organic dyes, in which short lifetimes are usually interfered with cellular autofluorescence^41^. A photochromic SP derivative was chosen as the fluorescence acceptor for its ability to undergo isomerization processes in response to a light stimulus^42^. By carefully varying the spectral overlap between the donor and the acceptor, the efficiency of the FRET process and consequently the fluorescence lifetime can be readily modulated.

**Fig. 1:**
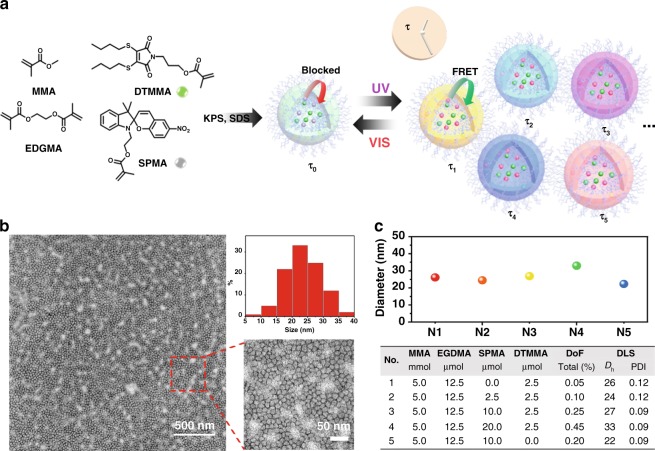
Fabrication of the photo-switchable polymeric nanogels. **a** Schematic representation of the preparation of light-triggered fluorescence lifetime switchable nanogels via emulsion polymerization; KPS: potassium persulfate; SDS: sodium dodecyl sulfate. **b** TEM micrographs of nanogel N4 (stained with 1 wt% uranyl acetate in water) illustrating the spherical morphology and histogram of observed size distributions; Scale bar: 500 nm (50 nm in zoomed region); Observed TEM sizes were collected from visualization of at least three separated samples; **c** Size distributions and corresponding hydrodynamic diameters of nanogels with variable SMPA and DTMMA ratios, obtained by DLS (detection angle = 173°) carried out in water at 25 °C (DoF = degree of functionalization, *D*_*h*_ = hydrodynamic diameter, PD = polydispersity). All determinations were repeated four times with 15 measurements recorded for each run.

Herein, methacrylate DTM and SP monomers were synthesized (Supplementary Figs. 1, 2)^43,44^ and copolymerized via one-pot micro-emulsion polymerization with methyl methacrylate (MMA) as the hydrophobic matrix and ethyleneglycol dimethacrylate (EGDMA) as the crosslinker (Fig. 1a)^45^. The experimental details are provided in the Methods section. In order to understand the effect of energy transfer on fluorescence lifetime properties, we constructed a series of crosslinked nanogels with different ratios of DTMMA and SPMA formed in situ during the polymerization (N1-5, Fig. 1c, Supplementary Table 1). The high conversion (>99%) of the polymerization was determined by monitoring the consumption of the acrylate groups in ^1^H NMR spectroscopy analysis of the nanogel (Supplementary Fig. 3). Thus, the amount of the two functional monomers in the resultant nanogels can be quantitatively assessed based on the starting ratio of two monomers. Transmission electron microscopy (TEM) visualization confirmed the spherical morphology of the nanogels (Fig. 1b and Supplementary Fig. 4) while the size was measured by dynamic light scattering (DLS) and ranged from 22 to 32 nm with a low polydispersity (Fig. 1c and Supplementary Fig. 5). The nanogel solutions were found to be stable for at least two months when stored in the dark (Supplementary Fig. 6a, b) and exhibited no obvious size change after 120 s of light irradiation (Supplementary Fig. 6c–f). The thermostability of the nanogel solution (N1 as an example) was also analysed by increasing the temperature from 25 to 70 °C in steps of 5 °C (Supplementary Fig. 6g, h). The hydrodynamic diameters of the nanogel undulated slightly within the range of 25–35 nm without visible sign of aggregation or disassembly.

### Evaluation of the energy transfer in the photoisomerization process

**Fig. 2:**
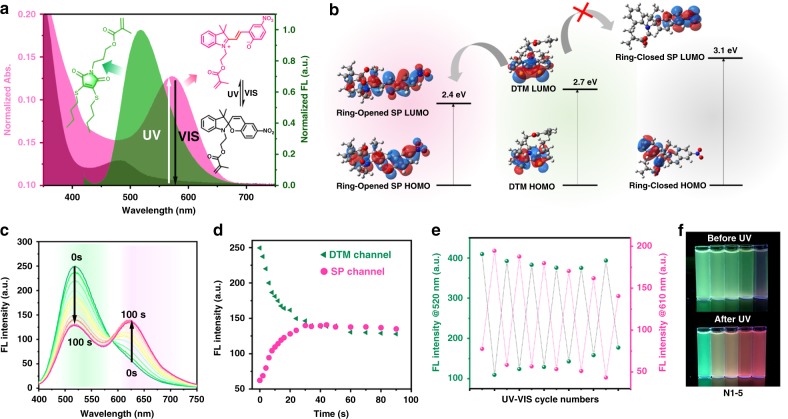
Energy transfer involved photophysical behavior of nanogels. **a** Spectra overlap between the emission of the DTMMA (donor) and absorption of the SPMA (open and closed form, acceptor); **b** Schematic illustration of HOMO and LUMO isosurfaces of DTMMA and SPMA with their first singlet excitation energies in a non-polar environment based on DFT and TD-DFT calculations; **c**, **d** Emission intensity changes of nanogel N4 before and after UV irradiation for 0–100 s (λ_ex. _= 410 nm, slit width: Ex. = 1 nm, Em. = 1 nm); **e** Fluorescence intensity cycling upon UV and vis irradiation (the DTM channel was recorded at 520 nm and the SP channel was recorded at 610 nm, irradiation time: 120 s); **f** Fluorescence images of the light-switchable nanogel solutions (N1–5) before and after UV irradiation for 120 s. Related data are provided in Source data file.

DTM, protected in the crosslinked polymeric environment, has a green emission in the range of 450–600 nm with high fluorescence quantum yield (Φ_f_ = 51%, 5-(6)-carboxyfluorescein as the reference) (Supplementary Figs. 7, 8). In comparison, the absorption of the ring-closed form of SP is negligible between 500 and 600 nm, while the ring-opened form of the photochromic dye absorbs in this wavelength range, offering the ideal scenario for an efficient FRET (Fig. 2a). The energy transferability between the donor and acceptor was quantitatively determined as the Förster radius (R_0_) which is the critical Förster distance for 50% FRET efficiency^46^. The critical transfer distance R_0_ of the DTM and ring-opened form of SP was estimated to be 18 Å (Supplementary Equations 9, 10, Supplementary Table 2).

To gain further insight into the energy transfer interactions between SP and DTM, time-dependent density functional theory (TD-DFT) calculations were carried out. The values of first singlet excited states for DTM and the ring-opened form of SP were calculated as 2.7 and 2.4 eV, respectively, while the first singlet excited state for the ring-closed form of SP was calculated as 3.1 eV, confirming that the FRET only occurs when SP is present as the ring-opened isomer (Supplementary Tables 3–7 and Fig. 2b).

### Photophysical behavior of nanogels using light as a stimulus

The 2D excitation-emission spectrum of the nanogel only containing DTM (N1) revealed that fluorescence emission of DTM was maintained in the nanogel environment with green emission (450–600 nm) when excited at ca. 410 nm (Supplementary Fig. 7). On the contrary, upon irradiation of the nanogels containing both DTM and SP (N2-4), the green emission in the DTM channel (λ_em._ = 520 nm) gradually decreased and a new peak around 610 nm emerged, indicating a FRET donor/acceptor pair was formed between the DTM and ring-opened SP (Fig. 2c). As anticipated, DTM emission could be fully recovered via irradiation with visible light, during which the FRET process is blocked by switching the SP back to the ring-closed form. The kinetics of FI against UV irradiation time, in N4, selected as an example, clearly revealed that the emission is closely related to the UV irradiation time (Fig. 2e). This can be ascribed to the increased amount of ring-opened SP during the light irradiation process. The photoconversion of SP reached a plateau after 31 s of irradiation, resulting in the final state in which the FRET is no longer influenced by UV (Supplementary Fig. 10). This can be fully recovered within 40 s of visible light irradiation, as confirmed by monitoring the intensity changing at 520 nm (DTM) and 610 nm (SP) (Fig. 2d and Supplementary Fig. 10). Moreover, the fluorescence emission was reversibly switched through alternating UV and visible irradiations (Fig. 2e). A slight decrease in intensity in the red channel and an increase in the green channel were observed after irradiating the sample with light 10 times presumably as a consequence of the irreversible photo-oxidation side reaction in SP molecules^42^. Different quenching effects on DTM emission in nanogels N2-4 were observed (Fig. 2f) after UV irradiation owing to the different efficiencies of the FRET process (Supplementary Figs. 15–18). A higher ratio of SP to DTM in the nanogels led to an enhanced quenching effect in the DTM channel as a result of the expanded spectral overlap and this provided a method by which the energy transfer efficiency and photoswitch contrast could be selectively controlled.

### Light-stimulated reversible fluorescence lifetime

Fluorescence lifetime imaging microscopy equipped with a time-correlated single-photon counting (TCSPC) was employed as a decoding technique for the visualization of localized fluorescence lifetime^31,47–49^. The determination of the intensity average lifetimes (τ_Av, I_) and amplitude average lifetimes (τ_Av, A_) was conducted using the Supplementary Equations 4, 5. The time-resolved fluorescence decay of the DTM nanogel (N1) was firstly measured in the time range of 1–100 ns excited by a pulsed laser (405 nm) with a calculated intensity-averaged lifetime (τ_Av, I_) of 28 ns (Supplementary Table 8). Nearly identical fluorescence decays were observed for N1 when changing the concentration (1–4 mg mL^−1^) or reversibly exposing the nanogel to UV and vis light (6 cycles), leading to a stable and sufficient lifetime platform (Supplementary Fig. 12).

**Fig. 3:**
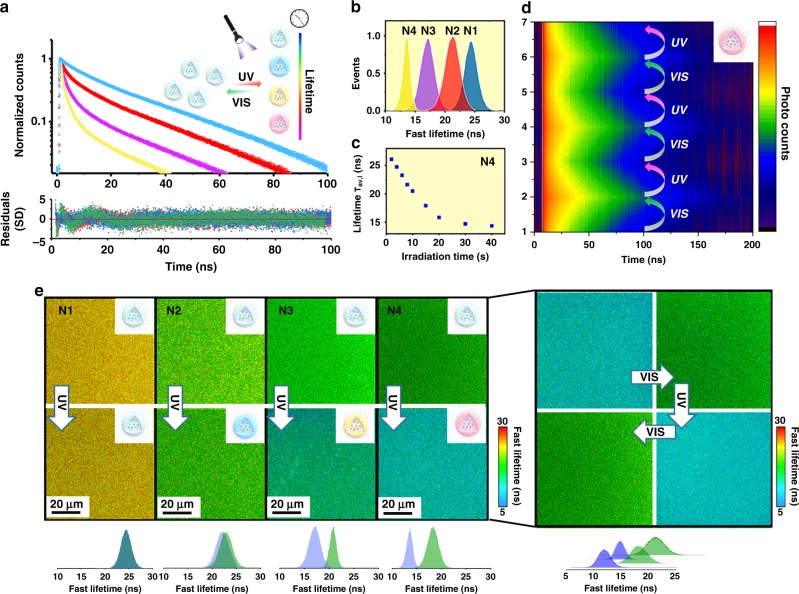
Quantification and visualization of the fluorescence lifetime in photo-switchable nanogels. **a** Fluorescence decay of nanogels (N1, blue, N2, red, N3, pink, N4, yellow) after UV irradiation for 120 s (λ_ex._ = 405 nm); Bottom: corresponding residuals. The sum of the three-exponential fitting with calculated averaged lifetime evaluation is given in Supplementary Information (Supplementary Table 8); **b** Fluorescence lifetime histogram of the nanogel solutions (N1-N4) after UV irradiation (λ_ex_ = 410 nm); **c** The average lifetime (τ_Av,I_) of nanogel solution (N4) after UV irradiating for 0–50 s; **d** Reversible 2D fluorescence decay of N4 upon alternating the UV and visible irradiation for six times (λ_ex._ = 375 nm, λ_em._ = 520 nm); **e** Fluorescence lifetime mapping of nanogel solutions (N1-4, 1 mg mL^−1^) before and after UV irradiation for 120 s (λ_ex._ = 405 nm, λ_em._ = 520 nm). Right: FLIM map showing the reversible lifetime of N4 in solution through alternating the UV and visible irradiation (λ_ex._ = 405 nm, irradiation time: 120 s). Color bar: Fast lifetimes (ns); Similar images were obtained for more than two sets of experiments.

Once this was established, the time-resolved fluorescence decays of the nanogels containing both DTM and SP (N2-4) were evaluated in situ after irradiating for 120 s to guarantee the photoconversion process. Two-dimensional time-resolved fluorescence decay spectra were firstly compared via monitoring the lifetime decay at different emission wavelengths (Supplementary Fig. 13). The decrease in the fluorescence lifetime of N4 solution in the DTM channel was observed, with a new fluorescence decay of ring-opened SP appearing from 600 to 650 nm, as the consequence of light-stimulated FRET. As depicted in Fig. 3a, b and Supplementary Table 8, the average lifetimes (τ_Av, I_) in N1-4 can be tuned from 15 to 28 ns after UV irradiation, linearly relating to the ratio of the two monomers. The FRET efficiency was calculated from the acquired lifetime results as 6% for N2 to 27% for N4 (Supplementary Table 9). Moreover, multiple lifetimes could be achieved in the same nanogel by tuning the UV irradiation time before reaching a plateau (Fig. 3c, Supplementary Fig. 14). However, with the aim to provide reproducible and quantitative outputs for barcoding applications, this study only focusses on manipulating the final states of the nanogel by changing the ratio of the FRET donor and acceptor. The overall decay of lifetime in N4 was fully reversible for 4 cycles of UV and vis light without any measurable alteration (Fig. 3d). This reversible behavior was further visualized through fluorescence lifetime imaging microscopy (FLIM), in which the different lifetimes can be observed (Fig. 3e, Supplementary Figs. 15–18).

Finally, we investigated the ability to encode information in our nanogels that could be subsequently decoded using UV light. Spherical PVA films were loaded with two nanogel solutions, N1 as the control, and N4 as representative of the photoswitchable system. While the lifetime decay of the two region-of-interest (ROIs) was comparable before irradiation, a change in lifetime after UV exposure was detected for the ROI corresponding to N4, while unchanged lifetime was observed for N1 (Supplementary Fig. 19). This demonstrates that not only information can be stored in our nanogel systems but can also be selectively decoded using light as a stimulus. Moreover, although the information can be visualized using both fluorescence microscopy and FLIM, a more quantitative result could be achieved with FLIM, which allows the lifetime to be accurately extracted by selecting ROIs.

### Reversible fluorescence lifetime barcoding in living cells

Having demonstrated the capability of dynamic lifetime barcoding in selective information encryption, we next investigated the ability of the reversible nanogel to be used as a fluorescence lifetime barcoding tool. In order to increase the stability of our nanogel system, the CLD was increased to 50% to achieve a denser core (N6). The cytotoxicity of the nanogels was firstly investigated in A549 cell line (cancer lung fibroblasts) where satisfactory cytocompatibility was observed when incubated with nanogels with or without fluorescent units up to 2 mg mL^−1^ (Supplementary Fig. 20). A549 cells were then incubated with nanogel N6 and the internalization of the material in these cells was evaluated. FLIM microscopy was employed to obtain quantitative photophysical values such as fluorescence lifetime, fluorescence count rate, and the total number of photons. From the FLIM images, nanogel N6 showed homogeneous distribution in the cell cytoplasm, while maintaining its photoswitchable properties (Supplementary Fig. 21).

**Fig. 4:**
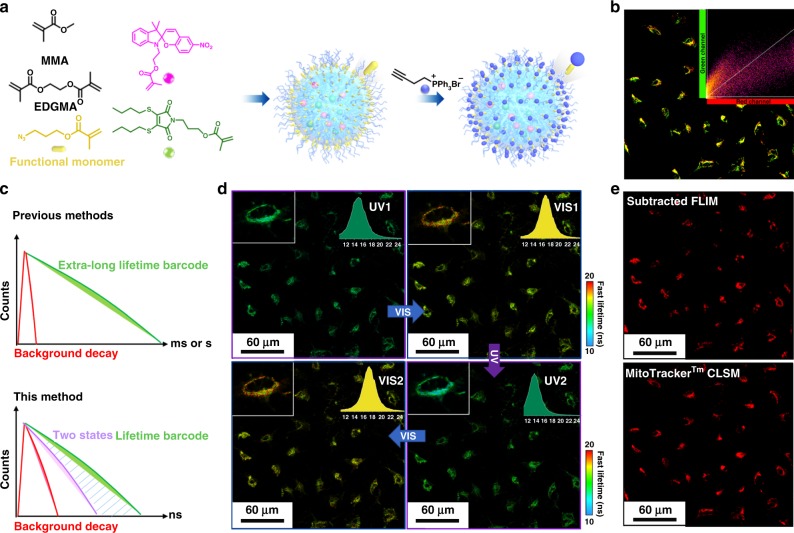
Functionalized photo-switchable nanogels for lifetime imaging in living cell. **a** Schematic illustration of the nanogel functionalization strategy and post-polymerization modification strategy for mitochondria tracking. **b** Confocal images of co-localized nanogel (TPP-N6, green channel) with MitoTracker Red (Red channel) in live A549 cells. Green channel: the ring-opened form of SP (λ_ex._ = 405 nm); Red channel: MitoTracker Red (λ_ex._ = 630 nm). The intensity correlation plots of TPP-N6 and MitoTracker Red were processed on imageJ and Pearson correlation coefficient was analyzed using Coloc2 plugins; **c** Schematic illustration of the lifetime amplification strategy using the reversible method compared with the previous method which required extra-long lifetime. **d** FLIM images of the nanogel with mitochondria tracker (TPP-N6) in live A549 cells under the stimulation of remote UV and Vis manipulation (Irradiation time: 120 s). Similar images were obtained for more than two sets of experiments. The insert images are the lifetime histograms of the nanogel in the whole image; Scale bar: 60 μm; Color bar: Fast lifetimes (ns). **e** The FLIM image (top) of TPP-N6 in live A549 cells before UV irradiation was subtracted with the same range after UV irradiation calculated by ImageJ. The subtracted image (top) was compared with the confocal fluorescence (bottom) of MitoTracker Red (Red channel).

We then sought to investigate the intracellular encoding and decoding process in more detailed cell structures, such as the mitochondria. In order to target mitochondria, triphenylphosphonium (TPP) was conjugated to the nanogel via an azide-alkyne cycloaddition to obtain TPP-N6, after functionalizing the nanogel with an azide reactive group (Fig. 4a, Supplementary Figs. 22–26)^50^. The functionalized nanogel was incubated in live A549 cells along with commercial MitoTracker Red for comparison. From confocal fluorescence analysis, co-localization was observed between the DTM channels in TPP-N6 and the red channel of commercial Mito Tracker (Supplementary Fig. 27), with a Pearson correlation coefficient (PCC) of 0.85 for TPP-N6 compared with the nanogel without the TPP modification (0.57), indicating the successful localization of these materials inside the mitochondria (Fig. 4b and Supplementary Fig. 28). Importantly, the incorporation of the tracker group did not influence the reversibility of the nanogel system.

Compared with traditional lifetime barcoding materials that require very long lifetimes (µs-ms) to minimize the effect of autofluorescence in the cellular environment, the reversible nature of the lifetime in this nanogel system provides a strategy to amplify the signal to noise ratio by deducting the two FLIM images between the reversible states (Fig. 4c). As an example, the FLIM image of TPP-N6 after UV irradiation (UV2, Fig. 4d) was deducted from the FLIM image before UV irradiation (Vis1, Fig. 4d), resulting in an amplified image in which the difference in lifetime was doubled (subtracted FLIM, Fig. 4e). In conclusion, the switchable nanogel system is not only able to track subcellular organelles with low toxicity but also provides a strategy to amplify the signal and diminish the background autofluorescence in FLIM without the need for the extra-long lifetimes.

## Discussion

Herein, we report a strategy to remotely control the fluorescence lifetime in polymeric nanogels via a photoisomerization-induced FRET process. The dynamic light-control over the fluorescence lifetime was successfully achieved via an efficient FRET process between the substituted maleimide DTM and a SP photoswitch, resulting in a series of nanogels with similar surface chemistry but broad dynamic lifetimes suitable for multiplex system coding and counting. These nanogel systems were further employed for lifetime barcoding using FLIM, where the multistates of the fluorescence were tracked and selectively visualized using a controllable, reversible, and non-invasive method. By simultaneously extracting lifetimes as the readout, the nanogel systems are capable of being selectively decoded with quantitative results, allowing information storage at the micro-scale. As a proof-of-concept, a mitochondrial tracker was introduced onto the nanogel via a click reaction obtaining live-cell lifetime barcoding at the subcellular scale and increasing the sensitivity of the imaging without the need for extra-long lifetimes.

We believe that the potential of the strategy presented herein will pave the way towards the application of soft materials in fluorescence lifetime barcoding. The multistate of the fluorescence lifetime in the polymeric nanogel has the potential to break the limitation of background influence without using metal ions. The spatially defined nature and multiplex-output of these nanogels will generate a high level of interest across a broad spectrum of areas and could lead to practical applications in bioanalytical science and bioengineering including high-throughput gene detection, clinical diagnosis, and drug screening.

## Methods

### Materials

All chemicals and reagents were purchased from either Sigma Aldrich, Fisher Chemicals, Acros Chemicals or Alfa Aesar. Solvents were purchased from Fisher Scientific and used as received. Dry solvents were used directly as obtained from a solvent tower purifying system. Commercially available monomer MMA and ethylene glycol dimethacrylate (EGDMA) were purified using a column of basic alumina prior to use. Experimental procedures for the preparation of azide functionalized monomer (3-azidopropyl methacrylate) were reported previously^51^. (But-3-yn-1-yl) triphenylphosphonium bromide was synthesized following previous literature^50^.

### Synthesis of dye-functionalized monomers

The 3-(3,4-bis(butylthio)-2,5-dioxo-2,5-dihydro-1H-pyrrol-1-yl)propyl methacrylate (DTMMA) and 2-(3′,3′-dimethyl-6-nitrospiro[chromene-2,2′-indolin]-1′-yl)ethyl methacrylate (SPMA)^43,44^ was synthesized through several reaction steps. Details of synthesis and characterization of the two functional monomers are given in the Supplementary methods and supplementary Figs. 30–35.

### Preparation of nanogel via micro-emulsion polymerization

Nanogels were synthesized by the micro-emulsion polymerization reported by our group^45^. In general, sodium dodecyl sulfate (0.1 g) was dissolved in water (50 mL) under N_2_ bubbling at room temperature. Subsequently, the methyl methacrylate (MMA, 0.5 g), ethylene glycol dimethacrylate (EGDMA 2.5 mg, CLD = 1%), DTMMA and SPMA were mixed before being added to the above solution under N_2_ protection. The reaction was kept stirring at 800 rpm and a solution of potassium persulfate (10 mg in 1 mL H_2_O) was added. The reaction was further stirred at 70 °C for 14 h. The resulting nanogels were filtered using a 0.45 μm nylon syringe filter and dialyzed (MWCO 3.5 kDa) against water prior to analysis. The final concentration was determined after freeze-drying.

Hydrodynamic diameters (*D*_h_) and size distributions (PD) of acquired nanogel were determined by DLS using a Malvern Zetasizer Nano ZS with a 4 mW He-Ne 633 nm laser module operating at 25 °C. Measurements were carried out at an angle of 173° and results were analyzed using Malvern DTS v7.03 software. All determinations were repeated four times with 15 measurements recorded for each run. *D*_h_ values were calculated using the Stokes-Einstein equation where particles are assumed to be spherical.

### Preparation of functionalized nanogels via click reaction

The azide functionalized nanogel was prepared using a similar method with minor changes. Briefly, sodium dodecyl sulfate (0.1 g) was added to water (50 mL) under N_2_ at room temperature. Subsequently, the MMA, EGDMA (0.125 g, CLD = 50%), DTMMA (1 mg), SPMA (8.4 mg) and azide monomer (3-azidopropyl methacrylate) were mixed before being added to the above solution under N_2_. The reaction was kept stirring at 800 rpm and a solution of potassium persulfate (10 mg in 1 mL H_2_O) was added. The solution was further stirred at 70 °C for 14 h. The resulting nanogels were filtered using a 0.45 μm nylon syringe filter and dialyzed (MWCO 3.5 kDa) against water prior to analysis. The final concentration was determined after freeze-drying.

TPP functionalized nanogels were prepared via an azide-alkyne copper-catalyzed click reaction^52^. The (But-3-yn-1-yl) triphenylphosphonium bromide was synthesized following a previous report^50^. Firstly, to the 50 mL solution (water/DMSO = 1:1) of azide functionalized nanogels (DoF 11%, 2 mg mL^−1^), sodium dodecyl sulfate (50 mg) was added under N_2_ at room temperature. Subsequently, (But-3-yn-1-yl) triphenylphosphonium bromide (50 mg), sodium ascorbate (5 mg) and CuSO_4_·5H_2_O (2.5 mg) were mixed before being added to the above solution under N_2_. The reaction was kept stirring at 60 °C for 24 h. The resulting nanogels were filtered using a 0.45 μm nylon syringe filter and dialysed (MWCO 3.5 kDa) against water for 2 days. The final concentration was determined after freeze-drying.

### Computational method

To obtain the most stable conformations of DTM and ring-closed SP, a Monte Carlo conformational search was carried out using the OPLS force field (for each system 1000 conformational search steps have been performed). The 20 low-energy structures were selected and re-optimized using the B3LYP and CAM-B3LYP functionals and the 6-311 G(d,p) basis set. It is worth noting that both DFT methods coincided with the same lowest-energy structures. Additional optimization processes were also performed using the M06-2X and PBE1PBE functionals and the 6-311 G(d,p) basis set. The dispersion effects (in exception of the M06-2X functional) and the solvent were included in all the optimization processes. The D3-Grimme’s dispersion with Becke-Johson damping factor was used to evaluate the dispersion effects. The solvent was considered using the polarization continuum model (PCM) and the dielectric constant of cyclohexane (ε = 2.0165). The ring-opened SP geometry was obtained from the modification of the lowest-energy ring-closed SP conformation. The harmonic vibrational frequencies were also calculated to verify that all the stationary points are minima of their potential energy surface.

These structures (DTM and ring-closed and ring-opened SP) were used for the TD-DFT calculations (B3LYP, CAM-B3LYP, M06-2×, and PBE1PBE) to describe the absorption and emission (geometry optimization of the first singlet excited state) processes. The Macromodel and Maestro software packages were used to carry out the conformational search. All the remaining calculations (geometry optimizations, frequencies, and TD-DFT) were performed using the Gaussian 16 program package (the references of the computational methods are reported in the Supplementary Information).

### Light irradiation

The UV (365 nm, 6 W) and light-emitting diode (White LED lamps, 2 W) were used as light sources for UV and visible light irradiation, respectively.

### Fluorescence steady-state and lifetime measurement and imaging

Steady-state fluorescence spectroscopy: All steady-state spectra were obtained with an Agilent Cary Eclipse Fluorescence spectrophotometer equipped with the photomultiplier tube (PMT) detector with a scan rate of 600 nm per minute. The emission kinetics were measured on an Edinburgh Instruments FS5 spectrofluorometer equipped with a Xenon lamp. The samples were measured in deionized water and the acquired data were analyzed in Origin 2019 (Origin Labs).

Fluorescence lifetime spectroscopy: Time-correlated single photon counting (TCSPC) was employed to obtain all fluorescence lifetime spectra. This was achieved with an Edinburgh Instruments FS5 spectrofluorometer equipped with 375 ± 10 nm ps pulsed diode laser source (PicoQuant) using 10 mm path length quartz cuvettes with four transparent polished faces (Starna Cells). The emission wavelength was chosen with a monochromator at 510 ± 4 nm. The signal level was kept below 5% of the light source repetition rate. Instrument response functions (IRF) were determined from the scatter signal solution of Ludox HS-40 colloidal silica (10% particles in water w/w). The analysis was performed on Fluoracle software (Edinburgh Instruments).

Fluorescence lifetime imaging microscopy (FLIM): FLIM was performed on LSM upgrade kit (PicoQuant) mounted on a FV3000 (Olympus) confocal microscope with a IX-81 inverted base (Olympus) and the 20× and 60× oil lens (Olympus) were used for imaging. The FV3000 system was driven with the FV31S-SW Viewer software platform (Olympus) with scan rates of 1 μs pixel^−1^ at 515 by 512 pixels. FLIM images and spectra were detected by single-photon avalanche diodes using a 520/60 bandpass filter (AHF analysentechnik) with a 405 nm (PicoQuant) pulsed diode laser driven at 2.5 MHz. FWHM for the 405 nm laser head was 59 ps and maximum power was 0.3 mW (attenuated by variable neutral density filters to prevent count pile up and maintain counting rates below 1% bin occupancy). Acquired images containing fluorescence lifetime were analyzed using fast-FLIM method implemented in SymPhoTime software (PicoQuant) and ImageJ. All IRF deconvolved exponential fits were performed with the number of exponents selected for completeness of fit as determined by boot-strap chi-squared analysis in SymPhoTime software, typically three.

### Cell culture and fluorescence imaging

Cell viability assay: A549 were purchased from Public Health England. Cells were cultured in F12K medium with addition of 10% FBS and 100 U mL^−1^ pen/strep at 37 °C and 5% CO_2_. Cells were seeded on 12-well plates at 2000 cells cm^−2^ and left to adhere and proliferate for 72 h. The medium was then replaced with the nanogel samples (N6 or nanogel without SPMA and DTMMA as control) in a concentration range from 0 to 2 mg mL^−1^. Briefly, a solution of nanogels in water (100 mg mL^−1^) was sterile filtered through a 0.45 μm filter. This solution was then diluted with cell culture medium (with the addition of 10% FBS and 100 U mL^−1^ pen/strep) to a final concentration of 10 mg mL^−1^. This stock solution was then used to prepare the dilutions directly on the well plates containing cells. After 24 h, the solution was removed and cells were washed with PBS (1 mL × 3) and incubated with 10% PrestoBlue viability assay following the supplier instructions. The FI was detected in a FluoStar Omega microplate reader (BMG Labtech) (λ_ex._ = 530 nm, λ_em._ = 590 nm). Cell data are reported as viability % in comparison to the control sample. Experiments were performed in triplicate.

Live-cell imaging and colocalization: A549 cells were cultured in F12K medium with addition of 10% FBS and 100 U mL^−1^ pen/strep at 37 °C and 5% CO_2_. Cells were seeded in glass-bottom micro dishes (Thermofisher Scientific) at 5000 cells cm^−2^ and incubated for 24 h at 37 °C in 5% CO_2_. After that, cells were pre-treated with commercial MitoTracker^TM^ Deep Red (20 μg mL^−1^) for 30 min prior to incubation with different nanogels at 1 mg mL^−1^ for one to two hours. Upon washing with cell medium three times, the resulting cells were transferred to an Olympus FV3000 confocal microscope equipped with an incubator to keep live cells at 37 °C in a 5% CO_2_ atmosphere during image acquisition. Live cells were imaged with a 60× oil-immersion objective with scan rates of 1 μs pixel^−1^ at 515 by 512 pixels both on Olympus FV3000 Microscopy for fluorescent images and PicoQuant LSM Upgrade Kit for fluorescence lifetime images. The original images were processed using CellSens software (Olympus), SymPhoTime 64 (PicoQuant) and ImageJ with Coloc2 plugins.

### Reporting summary

Further information on research design is available in the Nature Research Reporting Summary linked to this article.

## Supplementary information

Supplementary Information

Reporting Summary

## Data Availability

All data needed to evaluate the conclusions in the paper are present in the paper and/or the Supplementary Materials. The source data underlying Figs. 1c, 2c, 2d, 2e, 3a, 3b, 3c, and Supplementary Figs. 5, 6, 9, 10, 11, 12, 22, 26 are provided as a Source data file. All other data are available from the corresponding author.

## Source data

Source Data
