# Supplementary material for: Manipulating the fluorescence lifetime at the sub-cellular scale via photo-switchable barcoding
Source: Nat Commun. 2020 May 18;11:2460. doi: 10.1038/s41467-020-16297-3 (PMC7235003; doi:10.1038/s41467-020-16297-3)
Supplement: Supplementary file 1 — Supplementary Information [file 41467_2020_16297_MOESM1_ESM.pdf]

Supplementary Information

Manipulating the Fluorescence Lifetime at the Sub-Cellular Scale via Photo-Switchable Barcoding

Xie *et al.*

Supplementary Methods

Supplementary Method 1| General procedure for monomer synthesis.

All synthesized compounds were confirmed by ^{1}H-NMR and ^{13}C-NMR spectroscopy recorded on a Bruker Avance 300 or a Bruker Avance III HD 400 at 298k and 300, 400 MHz, respectively. Shifts (δ) are quoted in parts per million and quoted relative to an internal standard of trimethylsilane (TMS). High-resolution mass spectra were recorded on a Waters Xevo G2-XS QTof Quadrupole Time-of-Flight mass spectrometer.

Methyl 3,4-dibromo-2,5-dioxo-2,5-dihydro-1H-pyrrole-1-carboxylate (1).

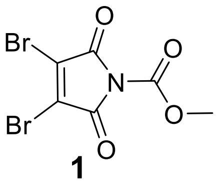

1

To a stirred solution of 3,4-dibromomaleimide (3 g, 11.76 mmol) in dry THF (40 mL) was added N-methyl morpholine (1.11g, 11.76 mmol). The solution was stirred for 15 min and then the methyl chloroformate (0.91 mL, 11.76 mmol) was added dropwise. The reaction was stirred under a nitrogen atmosphere for 40 min. Dichloromethane (200 mL) was added and the mixture was washed with water (200 mL × 3) and dried over anhydrous Na_2SO_4. The solvent was removed under reduced pressure and the crude product was dried without further purification.

3,4-dibromo-1-(3-hydroxypropyl)-1H-pyrrole-2,5-dione (2).

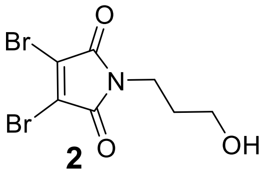

2

Methyl 3,4-dibromo-2,5-dioxo-2,5-dihydro-1H-pyrrole-1-carboxylate (2 g, 6.4 mmol) was dissolved in dry DCM (50 mL) and 3-amino-1-propanol (0.48 g, 6.7 mmol) was added. The reaction was stirred under a nitrogen atmosphere for 24 hours. Dichloromethane (150 mL) was added and the mixture was washed with NH_4Cl solution (100 mL), water (200 mL × 3) and dried over anhydrous

Na_2SO_4. The solvent was removed under reduced pressure and the product was purified by flash chromatography (3:1, *n*-hexanes: EtOAc) as a white solid. Yield: 73%.

^{1}H NMR (300 MHz, Chloroform-*d*) δ 3.78 (t, *J* = 6.6 Hz, 2H), 3.64 (t, *J* = 5.8 Hz, 2H), 1.93 – 1.78 (m, 2H). ^{13}C NMR (101 MHz, Chloroform-*d*) δ 164.3, 129.4, 59.3, 36.5, 31.0. HRMS(MaXis) m/z [M]: 310.8792, calculated 310.8793.

3,4-bis(butylthio)-1-(3-hydroxypropyl)-1H-pyrrole-2,5-dione (3)[1].

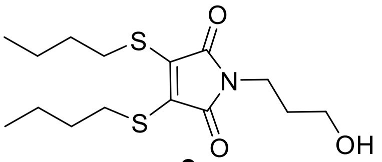

3

3,4-dibromo-1-(3-hydroxypropyl)-1H-pyrrole-2,5-dione (1 g, 3.2 mmol) and butanethiol (576 mg, 6.4 mmol) was dissolved in THF (50 mL) and cooled to 0 °C for 10 min. Then, triethylamine was added dropwise to the cooled solution and the reaction was stirred for 16 h at room temperature. Dichloromethane (150 mL) was added and the mixture was washed with water (200 mL × 3) and dried over anhydrous Na_2SO_4. The solvent was removed under reduced pressure and the product was purified by flash chromatography (2:1, *n*-hexane: EtOAc) as an orange oil. Yield: 56 %.

^{1}H NMR (300 MHz, Chloroform-*d*) δ 3.74 – 3.64 (m, 1H), 3.65 – 3.55 (m, 1H), 3.36 – 3.25 (m, 2H), 1.87 – 1.73 (m, 1H), 1.71 – 1.59 (m, 2H), 1.54 – 1.39 (m, 2H), 0.95 (t, *J* = 7.3 Hz, 3H). ^{13}C NMR (101 MHz, Chloroform-*d*) δ 167.2, 135.8, 59.0, 34.8, 32.5, 31.6, 31.3, 21.6, 13.8. HRMS(MaXis) m/z $[M+Na]^+$: 354.1171, calculated 354.1174.

3-(3,4-bis(butylthio)-2,5-dioxo-2,5-dihydro-1H-pyrrol-1-yl)propyl methacrylate (DTMMA).

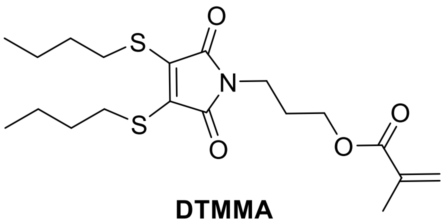

DTMMA

3,4-bis(butylthio)-1-(3-hydroxypropyl)-1H-pyrrole-2,5-dione (600 mg, 1.8 mmol) was dissolved in dry DCM (10 mL) and triethylamine (494 µL, 2.2 mmol) was added.

The reaction mixture was cooled to 0 °C for 10 min and the methacryloyl chloride (220 µL, 2.2 mmol) was added to the solution dropwise over 30 min. The solution was then stirred at room temperature overnight under a nitrogen atmosphere. 50 mL DCM was then added to the solution and followed by washing with water (50 mL × 3) and dried with anhydrous Na_2SO_4. The solvent was removed under reduced pressure to give orange oil that was purified by flash chromatography (4:1, hexane:EtOAc). Yield: 35 %.

^{1}H NMR (300 MHz, Chloroform-*d*) δ 6.10 (dt, *J* = 1.9, 0.9 Hz, 1H), 5.56 (t, *J* = 1.6 Hz, 1H), 4.15 (t, *J* = 6.1 Hz, 2H), 3.63 (t, *J* = 6.9 Hz, 2H), 3.28 (t, 4H), 2.05 – 1.96 (m, 2H), 1.68 – 1.54 (m, 4H), 1.52 – 1.37 (m, 4H), 0.93 (t, *J* = 7.3 Hz, 6H). ^{13}C NMR (101 MHz, Chloroform-*d*) δ 167.3, 166.6, 136.2, 135.7, 125.7, 61.9, 35.7, 32.5, 31.5, 27.6, 21.7, 18.3, 13.6. HRMS(MaXis) m/z $[M+Na]^+$: 422.1440, calculated 422.1436.

Compounds 1-3, SPMA were synthesized as reported in previous literature.[2, 3]

1-(2-hydroxyethyl)-2,3,3-trimethyl-3H-indol-1-ium iodide (4).

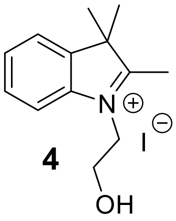

4

2,3,-trimethyl-3H indole (5 g, 31.5 mmol) and 2-iodoethanol (7.5 g, 44 mmol) was dissolved in MeCN (50 mL). The solution was refluxed under nitrogen for 24 hours. The reaction mixture was cooled to room temperature and product precipitated by the addition of hexane. The purple solid was filtered and dried without further purification. Yield: 95%.

9,9-dimethyl-2,3,9,9a-tetrahydrooxazolo[3,2-a]indole (5).

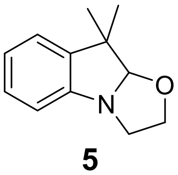

5

1-(2-hydroxyethyl)-2,3,3-trimethyl-3H-indol-1-ium iodide (7 g, 21 mmol) was suspended in degassed water (50 mL) under a nitrogen flow and potassium hydroxide (4 g, 70 mmol) was added. The solution was sonicated for 5 minutes

and stirred at room temperature for 30 min under a nitrogen atmosphere. Then the reaction mixture was extracted with diethyl ether (100 mL × 3). The combined organic layer was washed with brine and dried over anhydrous Na_2SO_4. The solvent was evaporated to afford an orange oil. Yield: 97%.

^{1}H NMR (300 MHz, Chloroform-*d*) δ 7.11 – 6.94 (m, 2H), 6.85 (td, *J* = 7.4, 1.0 Hz, 1H), 6.68 (dt, *J* = 7.9, 0.7 Hz, 1H), 3.83 – 3.72 (m, 1H), 3.70 – 3.58 (m, 1H), 3.58 – 3.37 (m, 2H), 1.35 (s, 3H), 1.31 (s, 3H), 1.11 (s, 3H). ^{13}C NMR (101 MHz, Chloroform-*d*) δ 150.5, 140.0, 127.5, 122.4, 121.7, 112.0, 109.0, 63.0, 50.1, 47.0, 28.1, 20.8, 17.6. HR-MS (MaXis) m/z found 203.1311, calculated 203.1310.

2-(3',3'-dimethyl-6-nitrospiro[chromene-2,2'-indolin]-1'-yl)ethan-1-ol (6)

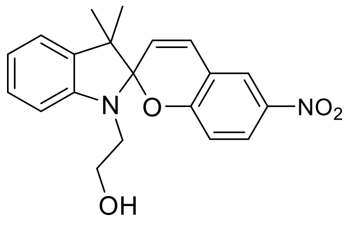

6

9,9-dimethyl-2,3,9,9a-tetrahydrooxazolo[3,2-a]indole (5 g, 16.7 mmol) and 2-Hydroxy-5-nitrobenzaldehyde (2.79 mg, 16.7 mmol) were dissolved in 20 mL ethanol. The reaction was left to reflux under nitrogen atmosphere for 4 h. The solvent was removed under reduced pressure to give a purple solid that was purified by flash chromatography (1:1, *n*-hexane:EtOAc) Yield: 89%.

^{1}H NMR (300 MHz, Chloroform-*d*) δ 8.00 – 7.86 (m, 2H), 7.13 (td, *J* = 7.6, 1.3 Hz, 1H), 7.03 (dd, *J* = 7.3, 1.3 Hz, 1H), 6.89 – 6.77 (m, 2H), 6.69 (d, *J* = 8.6 Hz, 1H), 6.60 (d, *J* = 7.8 Hz, 1H), 5.81 (d, *J* = 10.4 Hz, 1H), 3.82 – 3.60 (m, 2H), 3.39 (ddd, *J* = 14.9, 7.4, 5.4 Hz, 1H), 3.26 (dt, *J* = 14.9, 5.1 Hz, 1H), 1.65 (s, 1H), 1.22 (s, 3H), 1.13 (s, 3H). ^{13}C NMR (101 MHz, Chloroform-*d*) δ 159.3, 147.0, 135.9, 128.3, 127.9, 126.0, 122.8, 122.0, 121.9, 120.0, 118.6, 115.6, 106.9, 106.7, 60.9, 60.4, 52.9, 46.1, 25.9, 20.0. HRMS(MaXis) m/z $[M+H]^+$: found 353.1503, calculated 353.1501.

2-(3',3'-dimethyl-6-nitrospiro[chromene-2,2'-indolin]-1'-yl)ethyl methacrylate (SPMA)

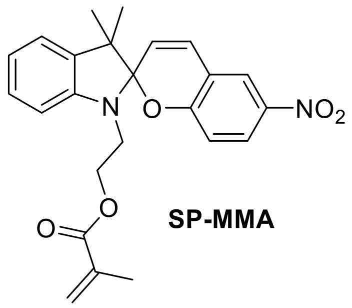

SP-MMA

2-(3',3'–dimethyl–6–nitrospiro[chromene-2,2'–indolin]-1'-yl)ethan-1-ol (2 g, 5.6 mmol) was dissolved in dry DCM (20 mL) in a dried flask under nitrogen and triethyl amine (1.4 mL, 6.7 mmol) was added. The reaction mixture was cooled to 0 °C for 10 min and then methacryloyl chloride (0.67 mL, 6.7 mmol) was added to the solution dropwise over 30 min. The solution was then stirred at room temperature overnight under a nitrogen atmosphere. 50 mL DCM was then added to the solution and followed by washing with water (100 mL × 3) and dried with anhydrous Na_2SO_4. The solvent was removed under reduced pressure to give a purple oil that was purified by flash chromatography (2:1, hexanes:EtOAc). Yield: 42%.

^{1}H NMR (400 MHz, Chloroform-*d*) δ 8.01 – 7.87 (m, 2H), 7.17 – 7.10 (m, 1H), 7.02 (dd, *J* = 7.3, 1.3 Hz, 1H), 6.86 – 6.80 (m, 2H), 6.66 (dd, *J* = 19.1, 8.2 Hz, 2H), 6.00 (t, *J* = 1.4 Hz, 1H), 5.80 (d, *J* = 10.3 Hz, 1H), 5.53 – 5.45 (m, 1H), 4.23 (t, *J* = 6.3 Hz, 2H), 3.55 – 3.24 (m, 2H), 1.85 (t, *J* = 1.3 Hz, 3H), 1.21 (s, 3H), 1.09 (s, 3H). ^{13}C NMR (101 MHz, Chloroform-*d*) δ 159.4, 146.7, 136.1, 135.7, 128.3, 127.9, 126.0, 126.0, 122.8, 121.8, 119.9, 118.4, 115.6, 106.8, 106.5, 62.7, 52.8, 42.4, 25.9, 19.8, 18.4. HRMS(MaXis) m/z $[M+H]^+$: 421.1760, calculated 421.1763.

Supplementary Method 2 | General procedure for fluorescence lifetime decay measurement and analysis.

Fluorescence lifetime image (FLIM) was collected using the LSM upgrade kit (PicoQuant) mounted on a FV3000 (Olympus) confocal microscope with a IX-81 inverted base (Olympus) and the 20x and 60x oil lens (Olympus) were used for imaging. FLIM images and spectra were detected by single-photon avalanche diodes using analyzed using the FLIM method implemented in SymPhoTime software (PicoQuant) and ImageJ. All IRF deconvolved exponential fits were performed with the number of exponents selected for completeness of fit as determined by boot-strap chi-squared analysis in SymPhoTime software, typically three. All IRF deconvolved exponential fits were performed with the 3 exponents selected for completeness of fit as determined by bootstrap Chi-square analysis in SymPhoTime software (PicoQuant) using the following equations:

Exponential model function:

$$\mathrm{S}(t) = \int_0^t \mathrm{E}(t')\mathrm{R}(t - t')\mathrm{d}t' \qquad (1)$$

S(t): Measured fluorescence decay;

E(t'): Measured instrumental response function;

R(t-t'): Theoretical sample decay model function;

$$\mathrm{R}(t) = \mathrm{A}_1\exp\left\{-\frac{t}{\tau_1}\right\} + \mathrm{A}_2\exp\left\{-\frac{t}{\tau_2}\right\} + \mathrm{A}_3\exp\left\{-\frac{t}{\tau_3}\right\} \qquad (2)$$

Reduced chi-square:

$$\chi^2 = \sum_k \mathrm{w}_\mathrm{k}^2 \frac{(\mathrm{F}_k - \mathrm{S}_\mathrm{k})^2}{n} \qquad (3)$$

W_k: Weighting factors for the individual data points.

S_k: The measurement data points.

F_k: The data points of the fitted curve.

N: The number of the free parameters which is approximately the number of fitted data points subtracted by the number of lifetime parameters used in the fit.

Intensity average lifetimes ($\tau_{Av,I}$) and amplitude average lifetimes ($\tau_{Av,A}$) were obtained from the fitting parameters according to the following equations:

$$\tau_{Av,I} = \frac{\sum A_i \tau_i^2}{\sum A_i \tau_i} \qquad (4)$$

$$\tau_{Av,A} = \frac{\sum A_i \tau_i}{\sum A_i} \qquad (5)$$

Supplementary Method 3| Computational methods

To obtain the most stable conformations of DTMMA and ring-closed SPMA, it was performed a Monte Carlo conformational search using the OPLS force field[4, 5] (for each system 1000 conformational search steps have been performed). The 20 low-energy structures were selected and re-optimized using the B3LYP[6, 7] and CAM-B3LYP[8] functionals and the 6-311G(d,p) basis set.[9, 10] It is worth noting that both DFT methods coincided with the same lowest-energy structures. Additional optimization processes were also performed using the M06-2X[11] and PBE1PBE[12] functionals and the 6-311G(d,p) basis set. The dispersion effects (in exception of the M06-2X functional) and the solvent were included in all the optimization processes. The D3-Grimme's dispersion[13] with Becke-Johson damping factor[14, 15] was used to evaluate the dispersion effects. The solvent was considered using the polarization continuum model (PCM)[16, 17] and the dielectric constant of cyclohexane (ε = 2.0165). The ring-opened SPMA geometry was obtained from the modification of the lowest-energy ring-closed SPMA conformation. The harmonic vibrational frequencies were also calculated to verify that all the stationary points are minima of their potential energy surface.

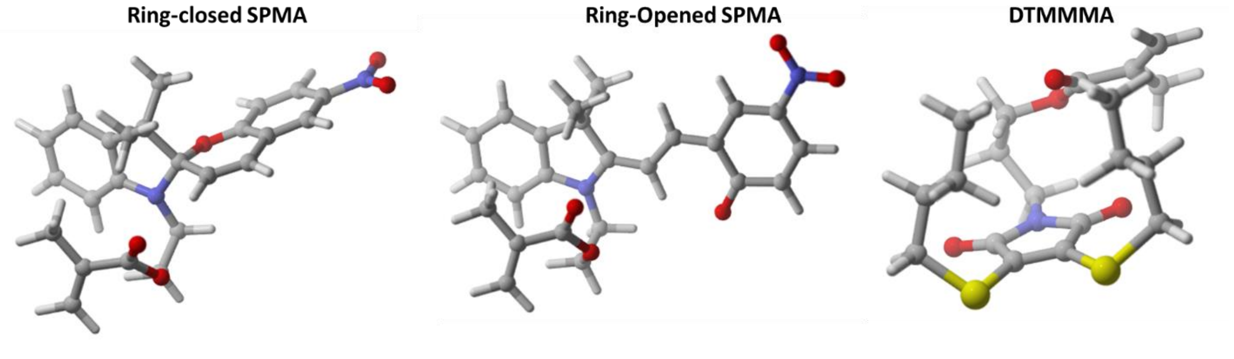

These structures (DTMMA and ring-closed and ring-opened SPMA) were used for the TD-DFT calculations[18, 19] (B3LYP, CAM-B3LYP, M06-2X, and PBE1PBE) to describe the absorption and emission (geometry optimization of the first singlet excited state) processes. In the TD-DFT

calculations, 50 excited states have been considered. The Macromodel[20] and Maestro[21] software packages were used to carry out the conformational search. All the remaining calculations (geometry optimizations, frequencies, and TD-DFT) were performed using the Gaussian 16 program package.[22]

The B3LYP and PBE1PBE functionals predict very similar excitation and emission energies (Supplementary Table S5 and S6). The lowest-energy absorption values correspond to HOMO → LUMO transitions, which mainly represent π bonding and π^* antibonding interactions. In DTMMA and ring closed SPMA systems, the π conjugation is mainly located on the dithiomaleimide ring and the phenyl of the indoline unit, respectively. Conversely, in the ring opened SPMA, the π conjugation is placed along almost all the structures. The photophysical parameters obtained from the CAM-B3LYP and M06-2X functionals (Supplementary Table S5 and S6) present a blue shift displacement between 50 and 100 nm with respect to the obtained B3LYP and PBE1PBE results. Nevertheless, all these four DFT functionals conclude that the FRET from DTMMA to the ring-closed SPMA is blocked and the only allowed FRET is to the ring-opened SPMA conformation.

Supplementary Tables

Supplementary Table 1 | Chemical composition and size characteristics of polymeric nanogel. The DoF (Degree of functionalization) and CLD (Cross-linking density of the EGDMA) were calculated by the following equations:

[a] Degree of functionalization of SPMA:

$$\mathrm{DoF(mol\%)} = \frac{[\mathrm{SPMA}]}{[\mathrm{MMA} + \mathrm{SPMA} + \mathrm{DTMMA}]} \times 100 \qquad (6)$$

[b] Degree of functionalization of DTMMA:

$$\mathrm{DoF(mol\%)} = \frac{[\mathrm{DTMMA}]}{[\mathrm{MMA} + \mathrm{SPMA} + \mathrm{DTMMA}]} \times 100 \qquad (7)$$

[c] Cross-linking density of EGDMA:

$$\mathrm{CLD(wt\%)} = \frac{2 \times \mathrm{Mass_{EGDMA}}}{\mathrm{Mass_{MMA}}} \times 100 \qquad (8)$$

No.	MMA mmol	EGDMA μmol	SPMMA		DTMMA		DoF	CLD[c]	DLS[d]	
			μmol	DoF[a]	μmol	DoF[b]	Total%	%	D_h (nm)	PD
1	5.0	12.5	0.0	0.00	2.5	0.05	0.05	0.01	26	0.12
2	5.0	12.5	2.5	0.05	2.5	0.05	0.10	0.01	24	0.12
3	5.0	12.5	10.0	0.20	2.5	0.05	0.25	0.01	27	0.09
4	5.0	12.5	20.0	0.40	2.5	0.05	0.45	0.01	33	0.09
5	5.0	12.5	10.0	0.20	0.0	0.00	0.20	0.01	22	0.09
6	5.0	625.0	20.0	0.40	2.5	0.05	0.45	0.50	23	0.05

[d] D_h and PD were average values measured 4 times from DLS analysis.

Supplementary Table 2| Photophysical data of FRET between DTM and ring-opened SP. J(λ) is the overlap integral of the fluorescence emission spectrum of the donor and the absorption spectrum of the acceptor was calculated as J(λ) which is dependent on the normalized donor fluorescence spectrum ($F_d(\lambda)$) and the molar extinction coefficient (M^{-1} cm^{-1}) of the acceptor ($\varepsilon_a(\lambda)$) according to the following equation (9):

R_o the critical Förster distance for 50% FRET efficiency, is defined by following equations.[23]

$$J(\lambda) = \int \varepsilon_a(\lambda) F_d(\lambda)\, \lambda^4 d\lambda \qquad (9)$$

$$\frac{R_0}{nm} = \left\{\frac{9\ln 10 \langle\kappa^2\rangle \phi_d J(\lambda)}{128\pi^5 n^4 NA}\right\}^{1/6}$$

$$= 0.02108\left[\frac{\kappa^2 \phi_d J(\lambda)}{n^4}\right]^{1/6} \qquad (10)$$

ε_a: The molar extinction coefficient of the acceptor ($M^{-1}cm^{-1}$). <κ^2>: The average squared orientational part of dipole-dipole interaction between donor and acceptor. Here, <κ^2> was assumed to be 2/3 which corresponds to the random orientation of donor and acceptor in the excited state. ϕ_d: The fluorescent quantum yield of the donor fluorophore, in this system; ϕ_d is the fluorescence quantum yield of the nanogel only containing DTM (N1) calculated *via* the relative method. *n*: refractive index. In this system, *n* is 1.45 for MMA. N_A: Avogadro constant (6.0221415×10^{23} mol^{-1}).

Donor	Acceptor	φ_d	J(λ) (M^{-1} $cm^{-1}nm^4$)	R_0 (nm)	$D_{effective}$ (nm)
DTMMA	SPMA	0.51	5.4E+12	1.8	≤3.6

Supplementary Table 3| TD-DFT excitation and emission energies, their corresponding oscillator strengths and the HOMO-LUMO gap of DTM, ring-opened and ring-closed SP structures at B3LYP-B3DJ/6-311G(d,p) level of theory using the PCM with ε = 2.0165.

	State	Major contribution	λ_{exc}[a]	f_{exc}[b]	Major contribution	λ_{em}[a]	f_{em}[b]	$\Delta\lambda$[a]	Δ_{L-H}[a]
**DTMMA**	1	H → L (70%)	453 (2.7)	0.100	H → L (70%)	574 (2.2)	0.079	121 (10.2)	369 (3.4)
	2	H-1 → L (62%)	360 (3.4)	0.019					
	3	H-3 → L (59%)	326 (3.8)	0.030					
**Ring-opened SPMA**	1	H → L (70%)	510 (2.4)	0.825	[c]				455 (2.7)
	2	H-1 → L (69%)	473 (2.6)	0.008					
	3	H-2 → L (70%)	373 (3.3)	0.389					
**Ring-Closed SPMA**	1	H → L (70%)	401 (3.1)	0.006	H → L (71%)	662 (1.9)	0.004	261 (4.7)	354 (3.5)
	2	H-1 → L (69%)	348 (3.6)	0.178					
	3	H -> L+1 (70%)	332 (3.7)	0.013					

[a] Values in nm and eV (in parenthesis). [b] Values in a.u. [c] The geometry of the first singlet excited state was not possible to converge.

Supplementary Table 4| TD-DFT excitation and emission energies, their corresponding oscillator strengths and the HOMO-LUMO gap of DTM, ring-opened and ring-closed SP structures at PBE1PBE-B3DJ/6-311G(d,p) level of theory using the PCM with ε = 2.0165.

	State	Major contribution	λ_{exc}[a]	f_{exc}[b]	Major contribution	λ_{em}[a]	f_{em}[b]	$\Delta\lambda$[a]	Δ_{L-H}[a]
**DTMMA**	1	H → L (70%)	438.2 (2.8)	0.108	H → L (70%)	558.6 (2.2)	0.092	120 (10.3)	335 (3.7)
	2	H-1 → L (60%)	347.9 (3.6)	0.015					
	3	H-3 → L (56%)	313.6 (4.0)	0.037					
**Ring-opened SPMA**	1	H → L (70%)	494.8 (2.5)	0.872	[c]				410 (3.0)
	2	H-1 → L (69%)	450.6 (2.8)	0.006					
	3	H-2 → L (70%)	356.6 (3.5)	0.372					
**Ring-Closed SPMA**	1	H → L (70%)	361.2 (3.4)	0.007	H → L (71%)	556.7 (2.2)	0.004	196 (6.3)	313 (4.0)
	2	H-1 → L (69%)	326.3 (3.8)	0.183					
	3	H → L+1 (69%)	313.2 (4.0)	0.015					

[a] Values in nm and eV (in parenthesis). [b] Values in a.u. [c] The geometry of the first singlet excited state was not possible to converge.

Supplementary Table 5| TD-DFT excitation and emission energies, their corresponding oscillator strengths and the HOMO-LUMO gap of DTM, ring-opened and ring-closed SP structures at CAM-B3LYP-B3DJ/6-311G(d,p) level of theory using the PCM with ε = 2.0165.

	State	Major contribution	λ_{exc}[a]	f_{exc}[b]	Major contribution	λ_{em}[a]	f_{em}[b]	$\Delta\lambda$[a]	Δ_{L-H}[a]
**DTMMA**	1	H → L (69%)	390 (3.2)	0.126	H → L (69%)	523 (2.4)	0.110	133 (9.3)	206 (6.0)
	2	H-1 → L (52%)	315 (3.9)	0.008					
	3	H-3 → L (48%)	286 (4.3)	0.037					
**Ring-opened SPMA**	1	H → L (70%)	464 (2.7)	1.053	H → L (69%)	534 (2.3)	0.721	76 (16.2)	250 (5.0)
	2	H-2 → L (64%)	383 (3.2)	0.002					
	3	H-1 → L (65%)	315 (3.9)	0.165					
**Ring-Closed SPMA**	1	H-6 → L (61%)	302 (4.1)	0.001	H-3 → L (71%)	532 (2.3)	0.000	230 (5.4)	199 (6.2)
	2	H-1 → L (68%)	289 (4.3)	0.199					
	3	H-1 → L+1 (56%)	275 (4.5)	0.042					

[a] Values in nm and eV (in parenthesis). [b] Values in a.u.

Supplementary Table 6| TD-DFT excitation and emission energies, their corresponding oscillator strengths and the HOMO-LUMO gap of DTM, ring-opened and ring-closed SP structures at M06-2X/6-311G(d,p) level of theory using the PCM with ε = 2.0165.

	State	Major contribution	λ_{exc}[a]	f_{exc}[b]	Major contribution	λ_{em}[a]	f_{em}[b]	$\Delta\lambda$[a]	Δ_{L-H}[a]
**DTMMA**	1	H → L (69%)	386 (3.2)	0.120	H → L (69%)	513 (2.4)	0.104	127 (9.7)	5.7
	2	H-4 → L (47%)	321 (3.9)	0.007					
	3	H-1 → L (48%)	293 (4.2)	0.029					
**Ring-opened SPMA**	1	H → L (70%)	471 (2.6)	1.049	H → L (69%)	565 (2.2)	0.678	94 (13.2)	4.5
	2	H-2 → L (62%)	378 (3.3)	0.003					
	3	H-1 → L (68%)	315 (3.9)	0.195					
**Ring-Closed SPMA**	1	H-8 → L (50%)	303 (4.1)	0.000	H → L+4 (66%)	557 (2.2)	0.000	254 (4.9)	6.0
	2	H-1 → L (67%)	283 (4.4)	0.121					
	3	H-1 → L+1 (54%)	272 (4.6)	0.081					

[a] Values in nm and eV (in parenthesis). [b] Values in a.u. [c] The geometry of the first singlet excited state was not possible to converge.

Supplementary Table 7| Cartesian coordinates (in Å) of the stationary points in the ground state optimized at the B3LYP-D3BJ/6-311G(d,p) level of theory in solution using the PCM model with ε = 2.0165.

DTMMA

C	-1.714546	1.868008	-0.576829
C	-2.069667	0.455815	-0.923160
C	-1.015826	-0.107556	-1.566129
C	0.094989	0.913640	-1.624583
N	-0.397007	2.059491	-1.001896
O	-2.389832	2.707860	-0.026727
O	1.205916	0.796190	-2.088958
S	-1.024799	-1.678348	-2.313987
S	-3.630363	-0.268699	-0.632359
C	0.750041	-2.183752	-2.332493
C	1.363271	-2.390313	-0.952988
H	1.302989	-1.448296	-2.909566
H	0.715000	-3.120220	-2.895044
C	0.720293	-3.497007	-0.116680
H	2.424843	-2.618260	-1.106922
H	1.340576	-1.453137	-0.394577
C	1.465902	-3.728899	1.198724
H	-0.320865	-3.233601	0.091680
H	0.691899	-4.425640	-0.699778
H	0.971196	-4.490190	1.807390
H	2.490956	-4.065315	1.012752
H	1.516870	-2.805976	1.780094
C	-3.929699	0.229139	1.126446
C	-2.777352	-0.089334	2.075646
H	-4.168879	1.289782	1.138589
H	-4.825493	-0.339331	1.387115
C	-2.334643	-1.552422	2.108087
H	-1.919294	0.542908	1.827868
H	-3.094657	0.220351	3.079007
C	-1.191762	-1.788910	3.096369
H	-2.021103	-1.856448	1.105655
H	-3.193467	-2.183889	2.364614
H	-0.896021	-2.840479	3.112140
H	-0.310579	-1.201718	2.825162
H	-1.484871	-1.509437	4.113415
C	0.383460	3.267056	-0.756821
C	0.564328	3.568485	0.736584
H	-0.103913	4.110517	-1.249690
H	1.346933	3.097266	-1.234377
C	1.021509	2.366814	1.539276
H	1.288270	4.382419	0.831535
H	-0.380181	3.915311	1.160694
H	0.285685	1.562999	1.530102
H	1.216294	2.631608	2.580472
O	2.246087	1.877272	0.948608
C	2.549585	0.579753	1.148649
O	1.871126	-0.162466	1.826213
C	3.800370	0.196122	0.427648
C	4.337896	-0.988290	0.725228
H	5.244664	-1.330622	0.240742
H	3.875511	-1.637139	1.457510
C	4.364740	1.149230	-0.587920
H	3.632316	1.334501	-1.377304
H	4.602191	2.113853	-0.133189
H	5.271078	0.738174	-1.034703

Ring-opened SPMA

C	2.940663	0.728137	1.003120
C	2.562414	1.877321	0.310144
C	3.515916	2.815781	-0.041972
C	4.851594	2.586117	0.308683
C	5.211813	1.436450	1.009979
C	4.257017	0.482824	1.372349
H	3.239218	3.712487	-0.583791
H	5.610828	3.307825	0.034369
H	6.248550	1.273336	1.277563
H	4.547433	-0.406502	1.915205
C	1.062018	1.855517	0.083905
C	0.678754	0.496104	0.688368

N	1.799329	-0.070658	1.219286
C	1.869474	-1.366454	1.883193
C	2.488873	-2.430741	0.975524
H	0.868206	-1.655994	2.187864
H	2.475294	-1.263451	2.787382
H	2.383055	-3.412443	1.436865
H	3.544543	-2.230881	0.805601
O	1.775600	-2.469741	-0.267104
C	2.221890	-1.781101	-1.358050
O	1.416974	-1.278438	-2.099392
C	3.691986	-1.749352	-1.651246
C	4.194446	-0.484797	-2.287827
H	3.610301	-0.259648	-3.182586
H	5.245522	-0.579969	-2.561138
H	4.087009	0.364030	-1.608074
C	4.431556	-2.847693	-1.485189
H	4.020648	-3.755633	-1.060126
H	5.467160	-2.876792	-1.803884
C	0.735973	1.943126	-1.421571
H	1.175453	2.856200	-1.828842
H	1.136989	1.084776	-1.958599
H	-0.338677	1.978788	-1.596815
C	0.404070	3.011439	0.872647
H	-0.679930	3.003832	0.760481
H	0.640826	2.941410	1.935836
H	0.782545	3.965048	0.499037
C	-0.559537	-0.120864	0.737603
C	-1.745214	0.433471	0.269176
H	-0.643125	-1.112389	1.153373
H	-1.716542	1.414510	-0.190579
C	-3.021213	-0.148705	0.330665
C	-3.282413	-1.472570	0.949696
C	-4.098584	0.585325	-0.219287
C	-4.671844	-1.915181	0.926627
C	-5.377787	0.095187	-0.198047
H	-3.917458	1.552641	-0.670006
C	-5.669934	-1.174968	0.382125
H	-4.868867	-2.881747	1.373716
H	-6.694596	-1.520034	0.373415
O	-2.397200	-2.174565	1.462698
N	-6.451213	0.879511	-0.772293
O	-7.589256	0.408329	-0.743045
O	-6.182715	1.978464	-1.261770

Ring-closed SPMA

C	-1.960876	1.070671	-0.658893
C	-1.626025	1.885586	0.429987
C	-2.281196	3.085792	0.629418
C	-3.280130	3.479324	-0.272317
C	-3.597014	2.668333	-1.357544
C	-2.939739	1.450911	-1.569857
H	-2.025623	3.719594	1.471266
H	-3.800290	4.418196	-0.127231
H	-4.363796	2.981234	-2.056689
H	-3.196163	0.834955	-2.421528
C	-0.558438	1.198704	1.261319
C	-0.068146	0.087221	0.250019
N	-1.205846	-0.108747	-0.622970
C	-1.084413	-0.994452	-1.767168
C	-2.280067	-1.933990	-1.882258
H	-0.179220	-1.586944	-1.634904
H	-0.978285	-0.427203	-2.700811
H	-2.118469	-2.655626	-2.682861
H	-3.198601	-1.386296	-2.079985
O	-2.402347	-2.703838	-0.672311
C	-3.077808	-2.207300	0.397842
O	-2.663009	-2.431987	1.510166
C	-4.384974	-1.506010	0.175163
C	-4.704915	-0.391501	1.129445
H	-4.591289	-0.735899	2.159518
H	-5.725248	-0.037485	0.980205
H	-4.023161	0.450012	0.986664
C	-5.244004	-1.978208	-0.730355
H	-4.993467	-2.809926	-1.377725
H	-6.239171	-1.559521	-0.826520
O	0.978929	0.686573	-0.606718

C	2.283776	0.398207	-0.455232
C	2.715382	-0.687825	0.332777
C	3.203560	1.198576	-1.137948
C	4.078743	-0.940612	0.439886
C	4.559476	0.941784	-1.022299
H	2.835652	2.017203	-1.742397
C	4.983692	-0.123278	-0.228537
H	5.293200	1.551928	-1.528494
C	1.693390	-1.519112	0.946083
H	2.002310	-2.434151	1.438764
C	0.401665	-1.186924	0.881331
H	-0.373000	-1.816445	1.299280
C	-1.198118	0.557621	2.508769
H	-1.669571	1.339580	3.107513
H	-1.955297	-0.180654	2.250805
H	-0.438892	0.066960	3.122358
C	0.579695	2.126471	1.694290
H	1.382391	1.557751	2.171099
H	0.992107	2.679112	0.851760
H	0.204876	2.844409	2.427148
H	4.443084	-1.768266	1.032398
N	6.415430	-0.394633	-0.102823
O	7.194626	0.331122	-0.713208
O	6.762768	-1.334220	0.606747

Supplementary Table 8| Photophysical decay and the exponential fitting of the nanogels before and after light illumination ($\lambda_{ex.}$ = 410 nm).*

	Parameter		Value /ns	Std. Dev. /ns	A %	χ^2	$\tau_{Av,I}$ /ns	Std. Dev. /ns	$\tau_{Av,A}$ /ns	Std. Dev. /ns
N1	**Vis**	1	31.10	0.06	0.63	1.005	28.4	0.052	22.1	0.019
		2	7.80	0.12	0.32					
		3	0.90	0.08	0.05					
	**UV**	1	32.00	0.22	0.52	1.085	28.3	0.048	19.0	0.017
		2	6.80	0.25	0.33					
		3	1.10	0.12	0.16					
N2	**Vis**	1	30.80	0.05	0.44	1.071	26.8	0.042	16.0	0.025
		2	6.24	0.02	0.38					
		3	1.17	0.02	0.19					
	**UV**	1	29.00	0.16	0.43	1.05	25.0	0.043	14.4	0.089
		2	6.00	0.15	0.31					
		3	1.31	0.04	0.26					
N3	**Vis**	1	28.00	0.075	0.34	1.095	23.4	0.042	11.8	0.039
		2	5.52	0.071	0.35					
		3	1.13	0.019	0.31					
	**UV**	1	25.40	0.051	0.19	1.205	19.0	0.043	7.0	0.035
		2	4.39	0.048	0.34					
		3	1.23	0.029	0.46					
N4	**Vis**	1	27.00	0.06	0.22	1.28	20.8	0.037	8.0	0.025
		2	4.82	0.039	0.34					
		3	1.08	0.009	0.44					
	**UV**	1	23.20	0.061	0.11	1.55	15.2	0.022	4.0	0.017
		2	3.22	0.046	0.33					
		3	0.82	0.019	0.46					

*Data was collected on the FLIM (PicoQuant) mounted on a FV3000 (Olympus) confocal microscope. 405 nm (PicoQuant) pulsed diode laser were applied as the excitation light source. The sample were measured in the aqueous solution at concentration 0.5 mg mL^{-1}.

Supplementary Table 9| Energy transfer efficiency in nanogel system with different ratio of DTM and SPMA. The energy transfer efficiency η_{ET} of different nanogel systems were calculated using the following equation[24]:

$$\eta_{ET} = 1 - {}^{\tau_{DA}}/_{\tau_D} \qquad (11)$$

where τ_D and τ_{DA} is the lifetime of the nanogel before UV irradiation and after UV irradiation at the different SP acceptor ratios, respectively.

[b] The energy transfer rate k_{ET} was calculated according to the equation (12).

$$k_{ET} = k_{total} - (k_r + k_{nr}) = \frac{1}{\tau_{DA}} - \frac{1}{\tau_D} \qquad (12)$$

k_{total} is the decay rate of the nanogel at 510 nm. k_r and k_{nr} is the radiative and non-radiative decay rate of the donor at 510 nm, respectively.

Nanogel	N1	N2	N3	N4	N5
$\eta_{ET}{}^{a}$	--	0.067	0.188	0.269	--
$k_{ET}{}^{b}$	--	0.003	0.009	0.017	--

Supplementary Figures

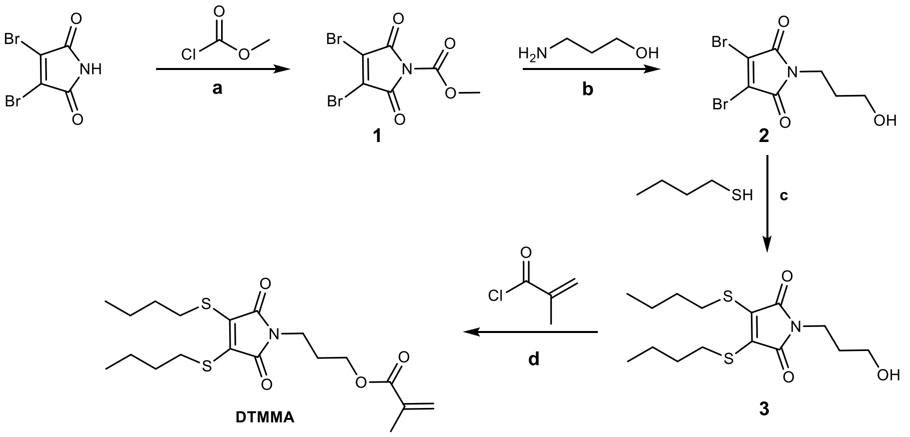

Supplementary Figure 1| Synthetic route for monomer DTMMA. a. N-methyl morpholine (NMM), THF, room temperature, 4 h; b. CH_2Cl_2, room temperature, overnight; c. Triethylamine, THF, room temperature, 16 h; d. Triethylamine, CH_2Cl_2, room temperature, overnight.

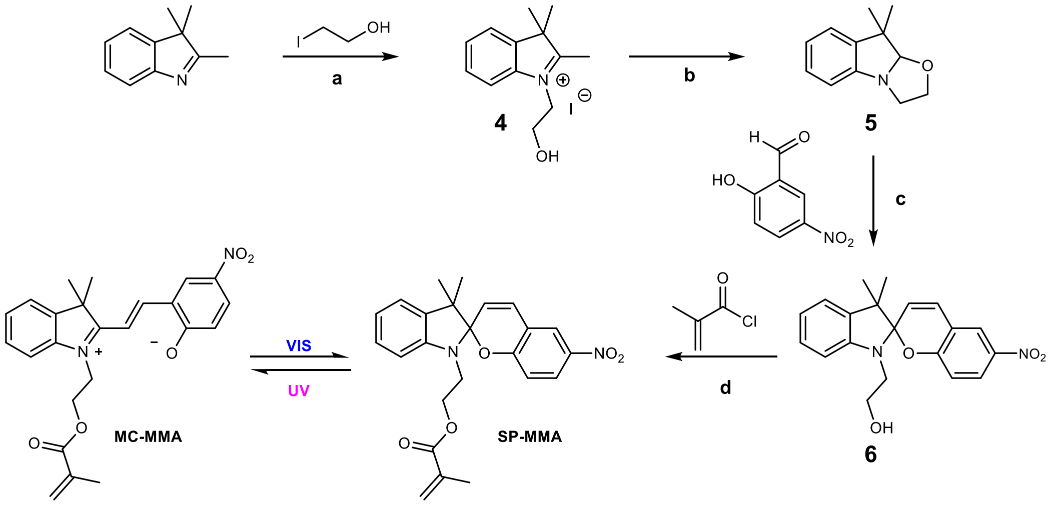

Supplementary Figure 2| Synthetic route for monomer SPMA. a. MeCN, reflux, 24 h; b. KOH solution, room temperature, 30 minutes; c. Ethanol, reflux, 4 h; d. Triethylamine, CH_2Cl_2, room temperature, overnight.

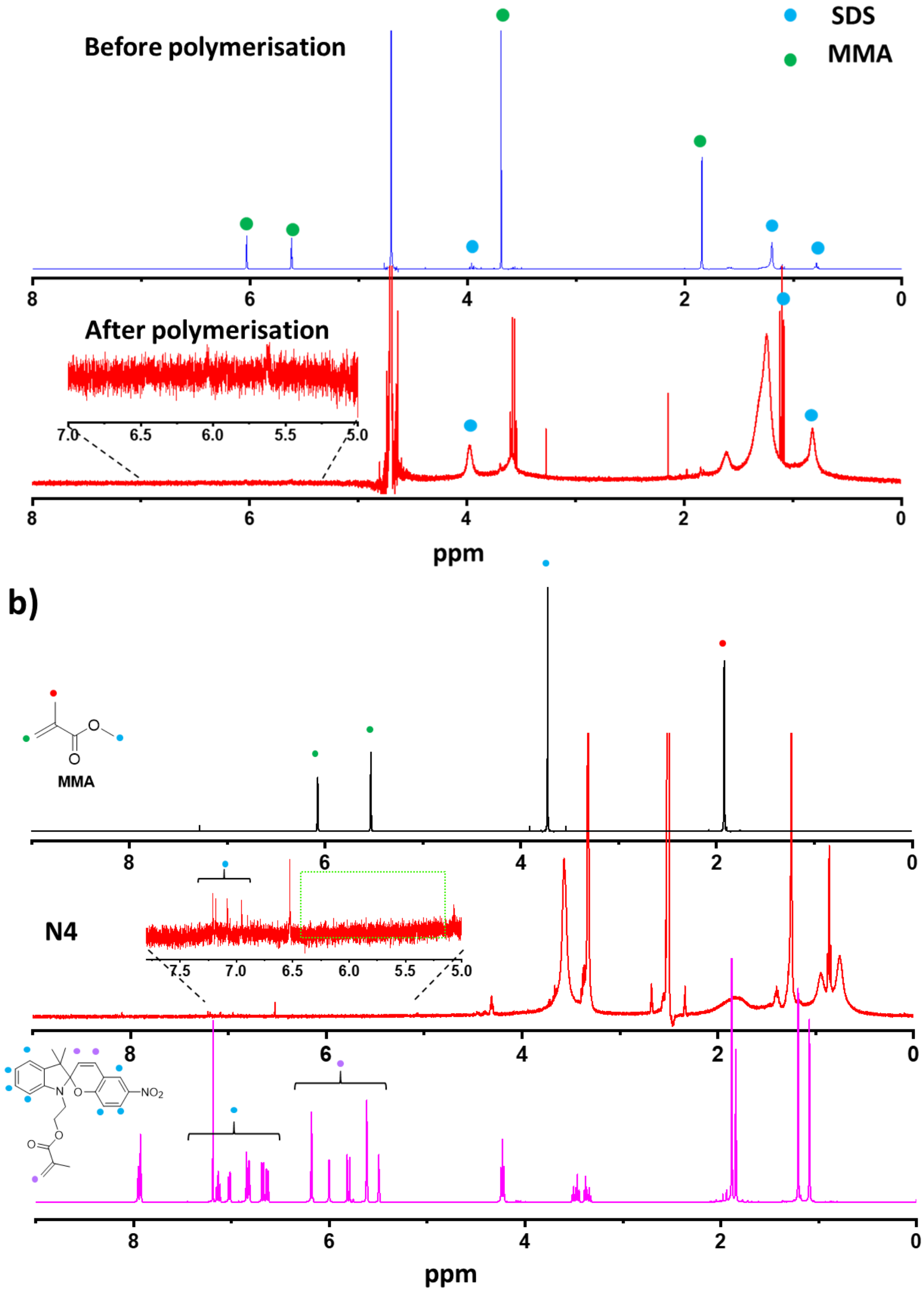

Supplementary Figure 3| a. ^{1}H NMR spectra monitoring the micro emulsion polymerization process in D_2O (400 MHz, 298K). b. Overlaid ^{1}H NMR spectra of nanogel N4 (red) in DMSO-d_6 with MMA (black) and SPMA (pink). The consumption of the acrylate groups around 5.5-6.3 ppm (inset) indicates full monomer conversion (400 MHz, 298K).

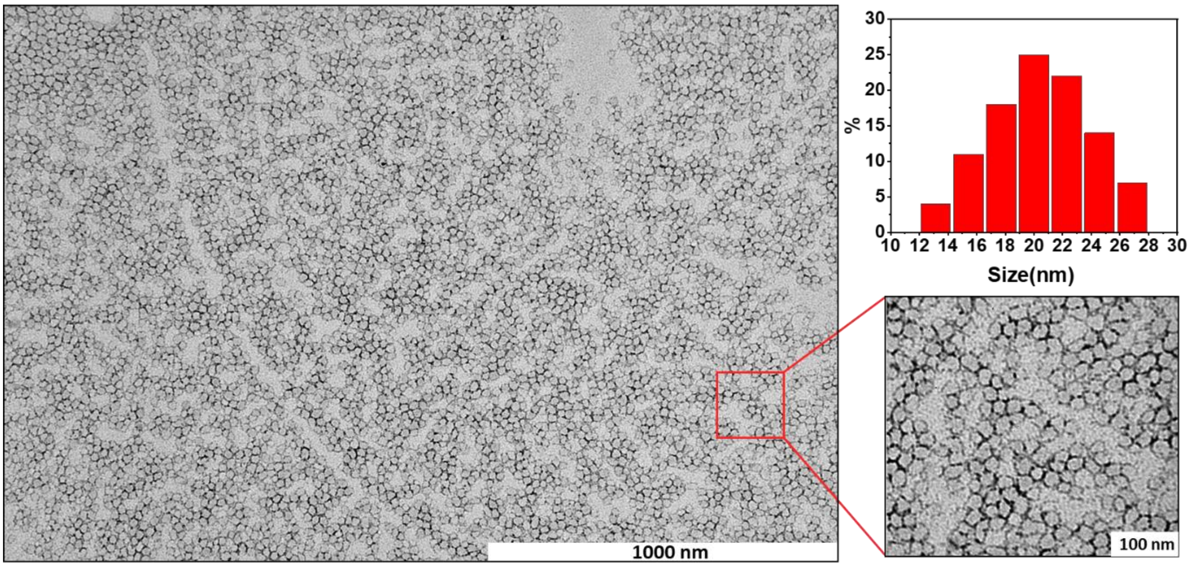

Supplementary Figure 4| TEM image of nanogel (N1) showing spherical morphology, stained with 1 wt% UA solution. The right image shows the statistical analysis of the nanogel size distribution (top) and the enlarged TEM (bottom). Dry-state stained transmission electron microscopy (TEM) imaging was performed on a JEOL JEM-1400 microscope operating at an acceleration voltage of 80 kV. All dry-state samples were diluted with deionized water to appropriate analysis concentration (0.01 mg mL^{-1}) and then deposited onto formvar-coated copper grids. After roughly 1 min, the excess sample was blotted from the grid and the grid was stained with an aqueous 1 wt% uranyl acetate (UA) solution for 1 min prior to blotting, drying and microscopic analysis. TEM observations were measured from more than three samples

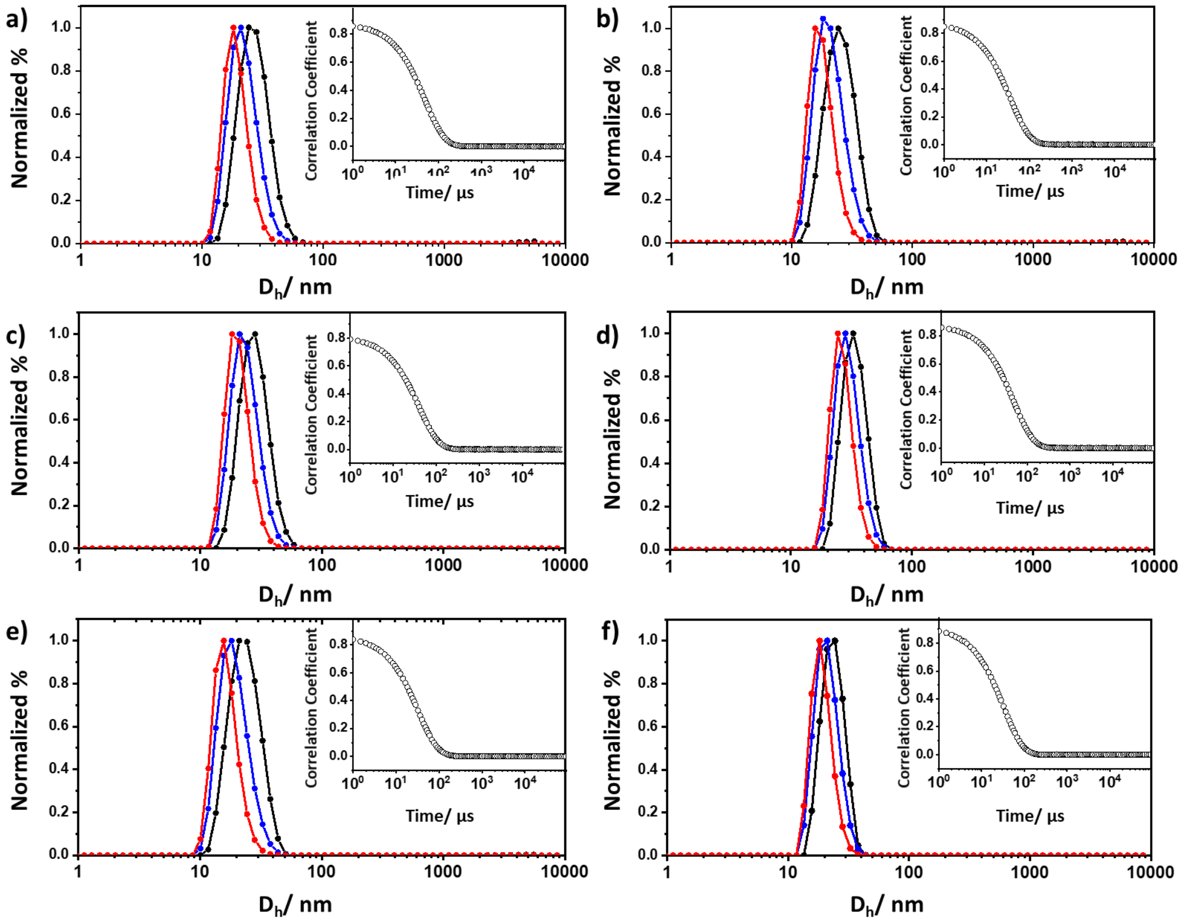

Supplementary Figure 5| a-f. Size distribution and corresponding hydrodynamic diameters obtained of N1-6 at 1 mg mL^{-1}, obtained by DLS (detection angle = 173°) and the corresponding autocorrelation function (inset). PD: Dispersity, D_h: hydrodynamic diameters, red: number weighted, blue: volume-weighted, black: intensity weighted. All determinations were repeated 4 times with 15 measurements recorded for each run. D_h values were calculated using the Stokes-Einstein equation where particles are assumed to be spherical.

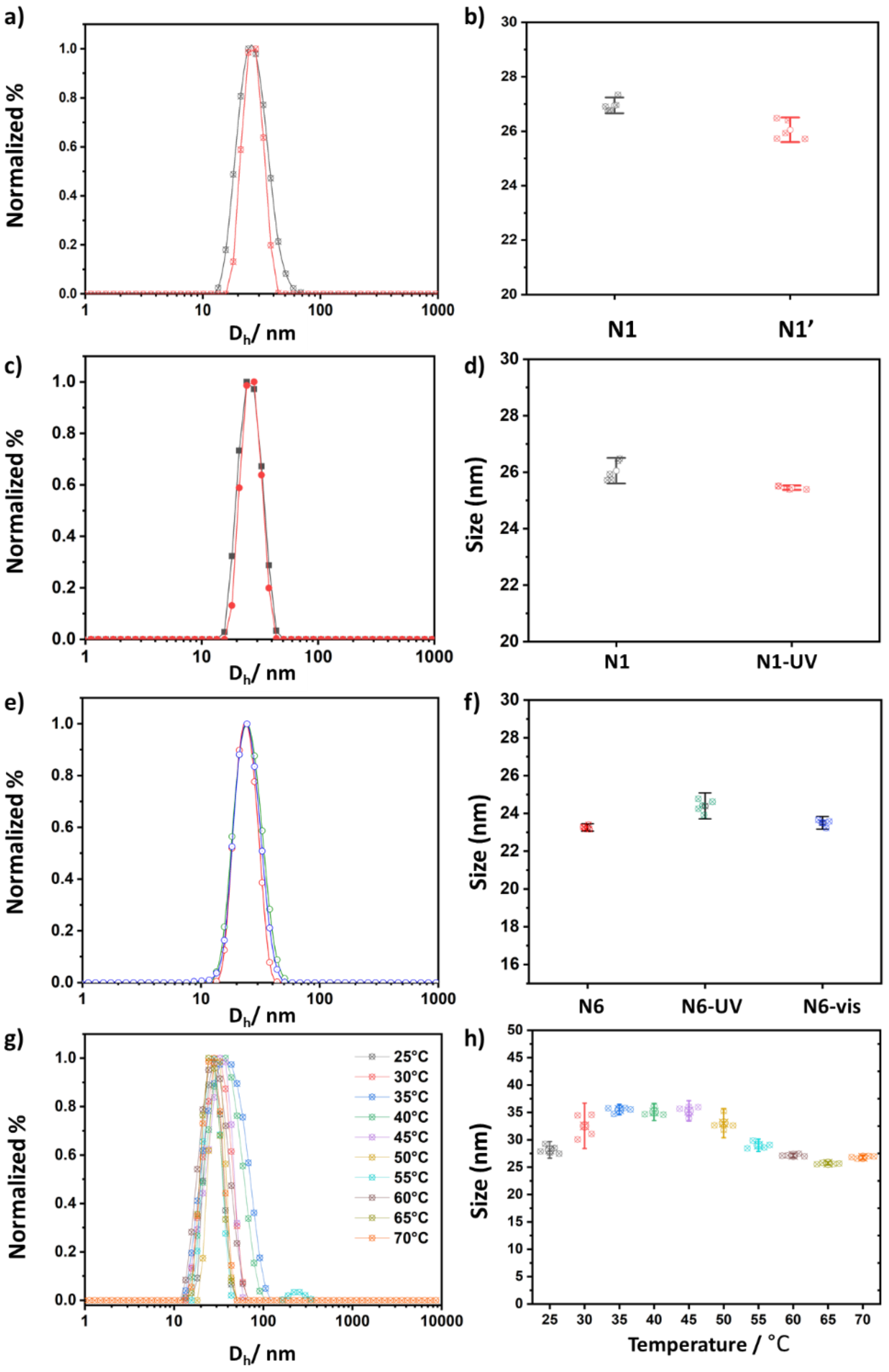

Supplementary Figure 6| a. Size distribution and b. corresponding hydrodynamic diameters of nanogel N1 before (black) and after (red) two months storage; c. Size distributions and d. corresponding hydrodynamic diameters of N1 before (black) and after (red) UV light irradiation for 120 s; e. Size distributions and f. corresponding hydrodynamic diameters of N6 after UV (green) and visible light (blue) irradiation for 120 s; g. Size distributions and h. corresponding hydrodynamic diameters of N1 at different temperatures. All Size distribution and corresponding hydrodynamic diameters were collected by DLS measurements (25-70 °C with steps of 5 °C), repeated 4 times with 15 measurements recorded for each run. Distribution data are presented as mean values +/− S.D. All error bars represent standard deviation (n = 4).

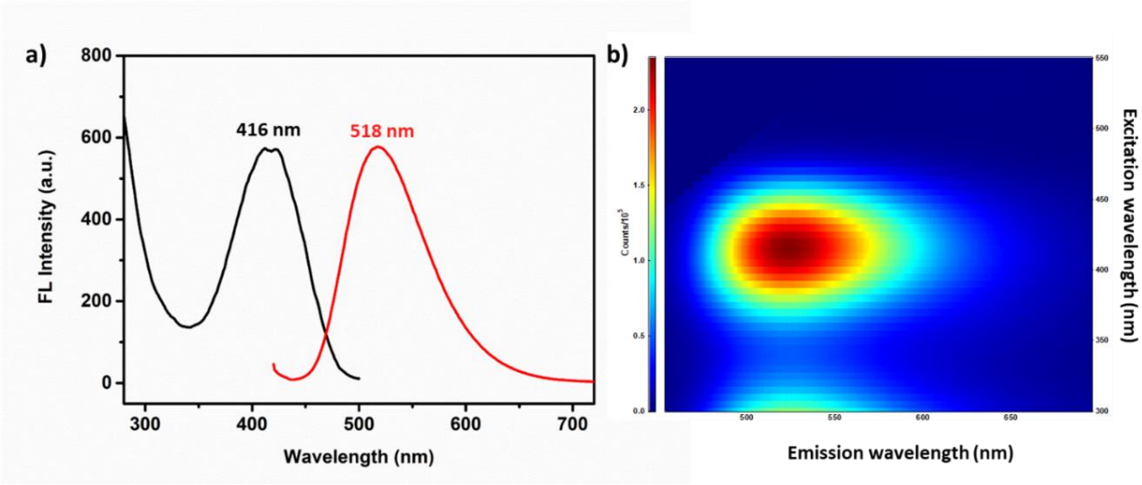

Supplementary Figure 7| a. Excitation and emission spectra of N1 in water at 0.5 mg mL^{-1} (slit width: Ex. = 1 nm, Em. = 1 nm); b. 2D excitation-emission spectra (with a 5 nm step) of N1 at 0.5 mg mL^{-1} (slit width: Ex. = 1 nm, Em. = 1 nm).

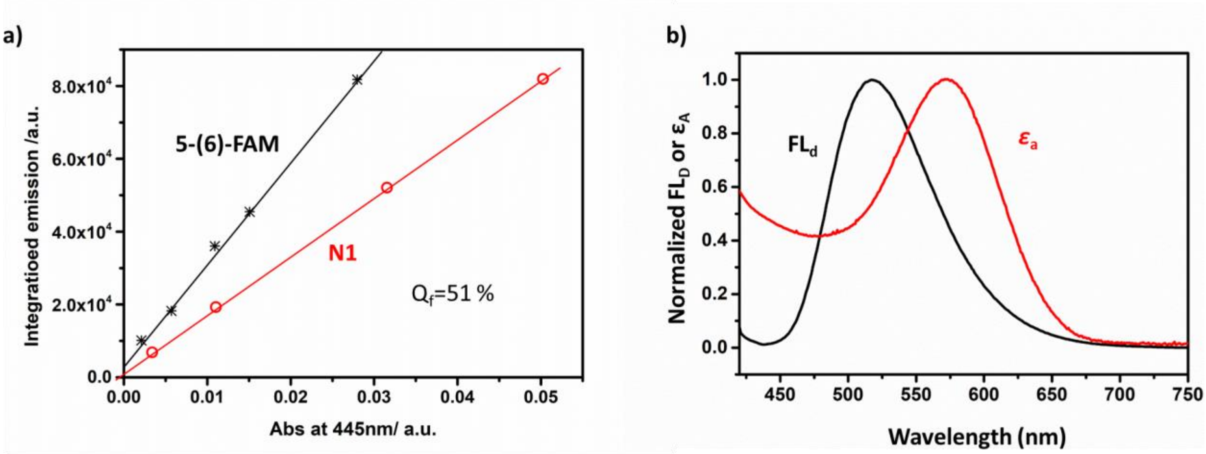

Supplementary Figure 8| Estimation of Relative Quantum Yield of the nanogel N1. a. Linear fit of the integrated emission of N1 and 5-(6)-FAM with absorption at 445 nm; b. Fluorescent emission of N1 (black line) and the molar extinction coefficient of SPMA (red line).The relative quantum yield (φ_d) of nanogel only containing DTM (N1) in aqueous solution was calculated using 5-(6)-carboxyfluorescein (5(6)-FAM) as the standard reference (φ_{st}=92%). The emission of both 5(6)-FAM and N1 were excited at 445 nm and the integration of the emission was liner fitted with the absorption at the same wavelength. By comparing the slope of the reference and N1, the relative quantum yield of N1 was calculated as 51% in aqueous solution.[25]

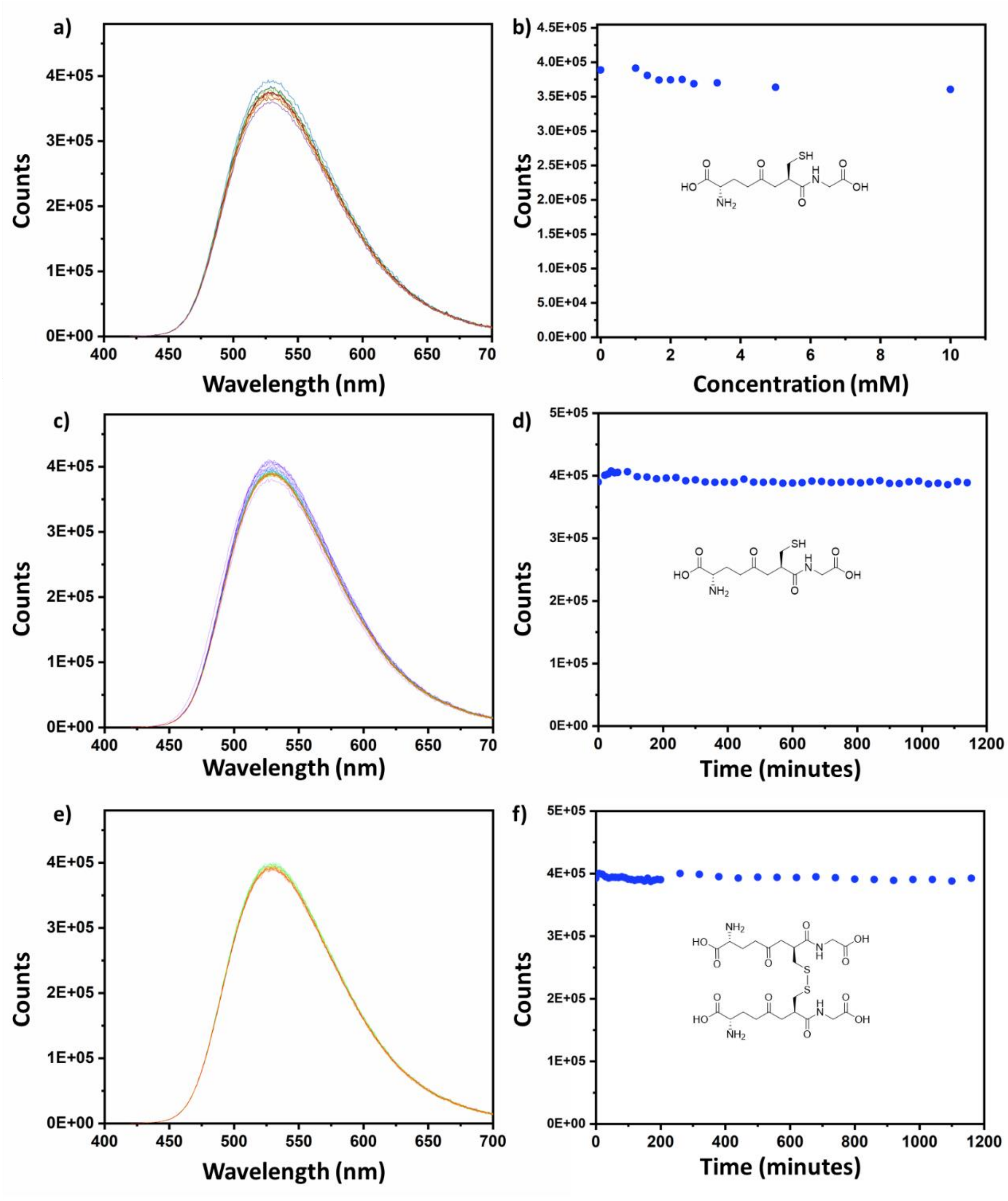

Supplementary Figure 9| a-b. Fluorescence emission and intensity of nanogel N1 (50% CLD) in different concentrations of reduced GSH; b) Fluorescence emission and intensity of N1 (50% CLD) in the presence of reduced GSH (10 mM) recorded for a total of 20 h; e-f. Fluorescence emission and intensity of N1 (50% CLD) in the presence of oxidized GSH (10 mM) recorded for a total of 20 h; (λ_{ex} = 410 nm, λ_{em} = 530 nm, C_{N1} = 0.5 mg mL^{-1}).

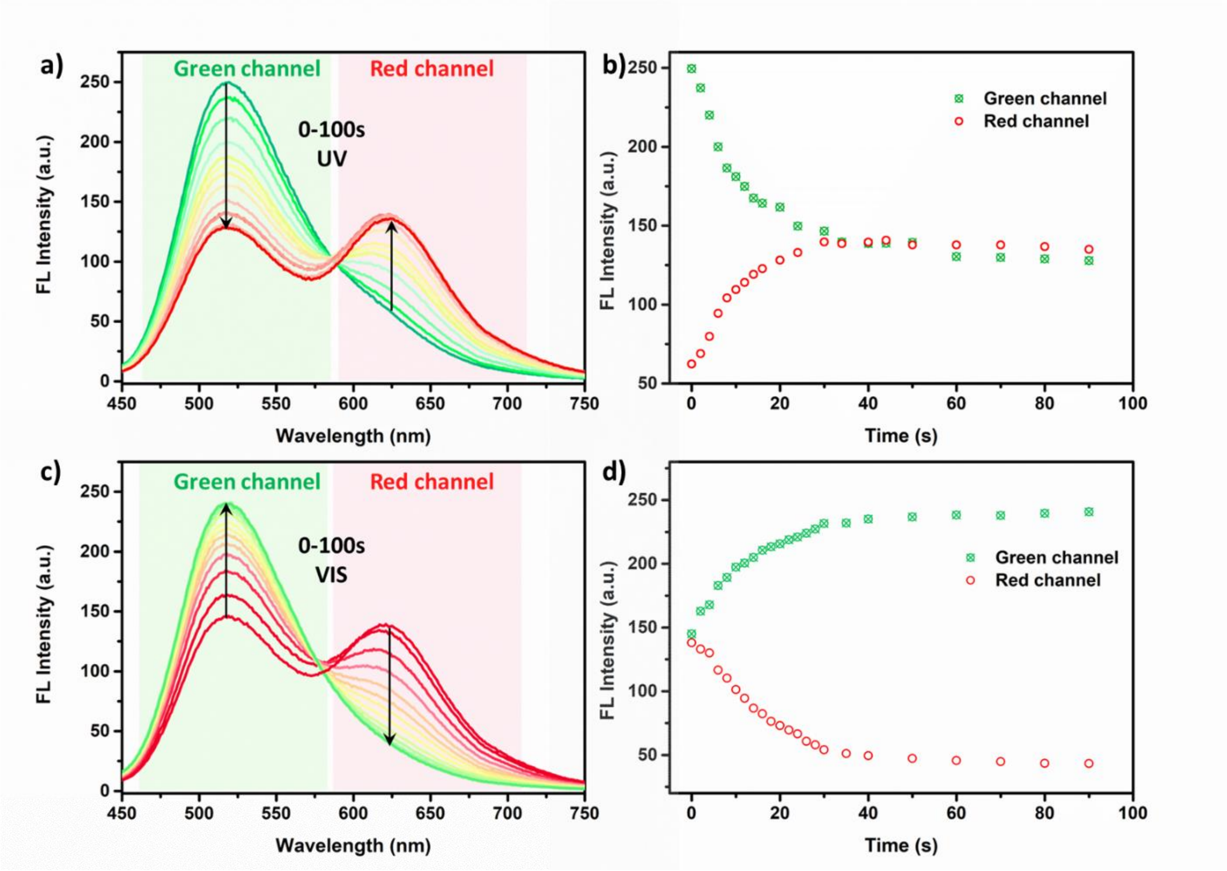

Supplementary Figure 10| a. Emission spectra of nanogel N4 after UV light irradiation for 0-100 s (λ_{ex}=410 nm, slit width: Ex. = 1 nm, Em. = 1 nm); b. Emission intensity changes at DTM channel (510 nm) and SP channel (610 nm) after UV light irradiating for 0-100 s c. Emission spectra of nanogel N4 after visible light irradiation for 0-100 s (λ_{ex}=410 nm, slit width: Ex. = 1 nm, Em. = 1 nm); d. Emission intensity changes at DTM channel (510 nm) and SP channel (610 nm) after visible light irradiating for 0-100 s (λ_{ex}=410 nm, slit width: Ex. = 1 nm, Em. = 1 nm; $C_{\mathbf{N4}}$=0.5 mg mL^{-1}).

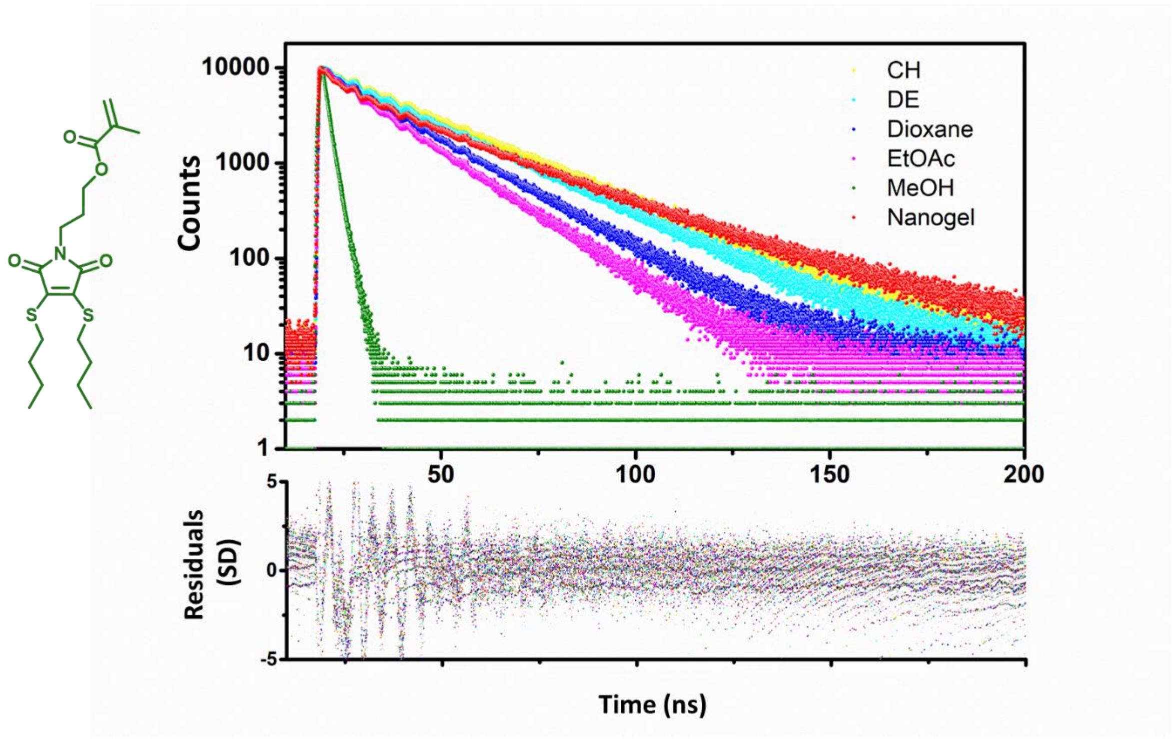

Supplementary Figure 11| Fluorescence lifetime decay of DTMMA small molecule in the different solvent with fitting and residuals (bottom). CH: cyclohexane, DE: diethyl ether, EtOAc: ethyl acetate, MeOH: methanol, Nanogel: N1 (λ_{ex} = 375 nm, λ_{em} = 510 nm, Slit: Ex. = 1, Em. = 1).

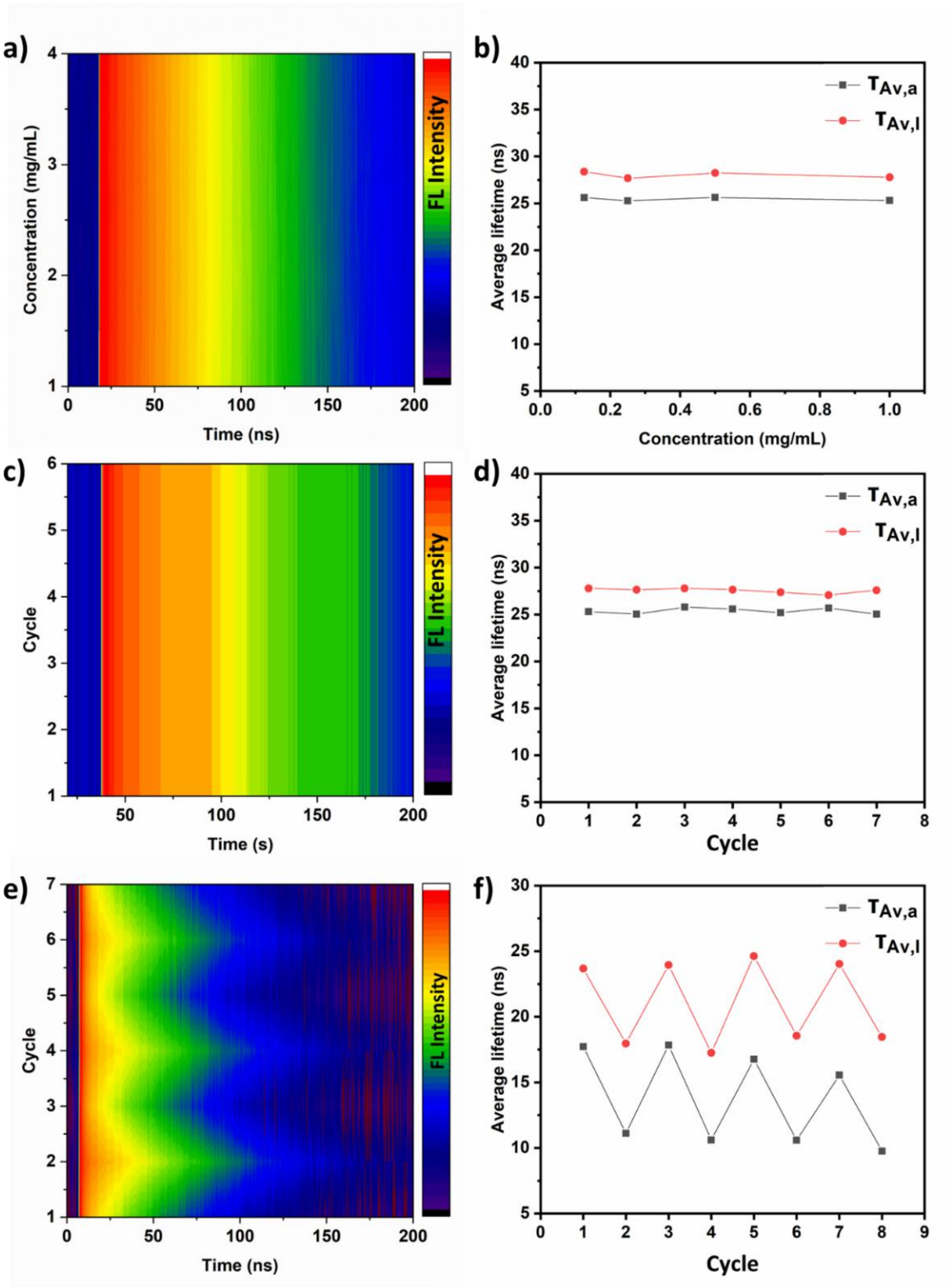

Supplementary Figure 12| a. Fluorescence lifetime decay and b. average fluorescence lifetime of N1 at different concentrations (0.1-1 mg mL^{-1}) in water monitored under excitation with a pulsed laser (λ_{ex} = 375 nm). c. Fluorescence lifetime decay and d. average fluorescence lifetime of N1 upon alternating UV (120 s) and visible light (120 s) irradiation for 7 times (λ_{ex} = 375 nm, C_{N1} = 0.5 mg mL^{-1}); e. Fluorescence lifetime decay and f) average fluorescence lifetime of N4 upon alternating UV (120 s) and visible light (120 s) irradiation for 8 times (λ_{ex} = 375 nm, $C_{\mathbf{N4}}$ = 0.5 mg mL^{-1});

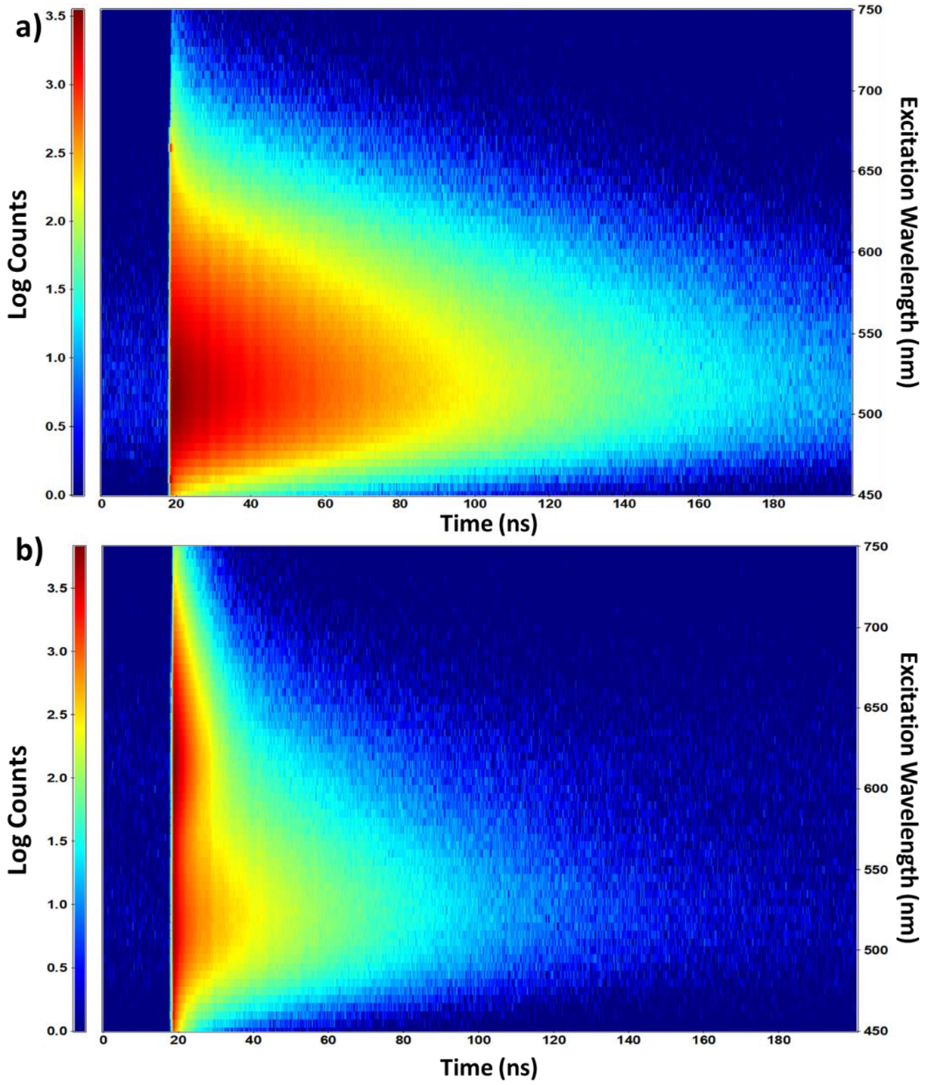

Supplementary Figure 13| a. 2D Fluorescence lifetime decay of N1 after UV irradiation (120 s); b. 2D Fluorescence lifetime decay of N4 after UV irradiation (120 s); 2D Fluorescence lifetime was recorded under the continuous excitation of the nanogel solution (C = 0.5 mg mL^{-1}) with a pulsed laser (λ_{ex} = 375 nm). Fluorescence lifetime decays were monitored at different emission wavelengths with 5 nm steps.

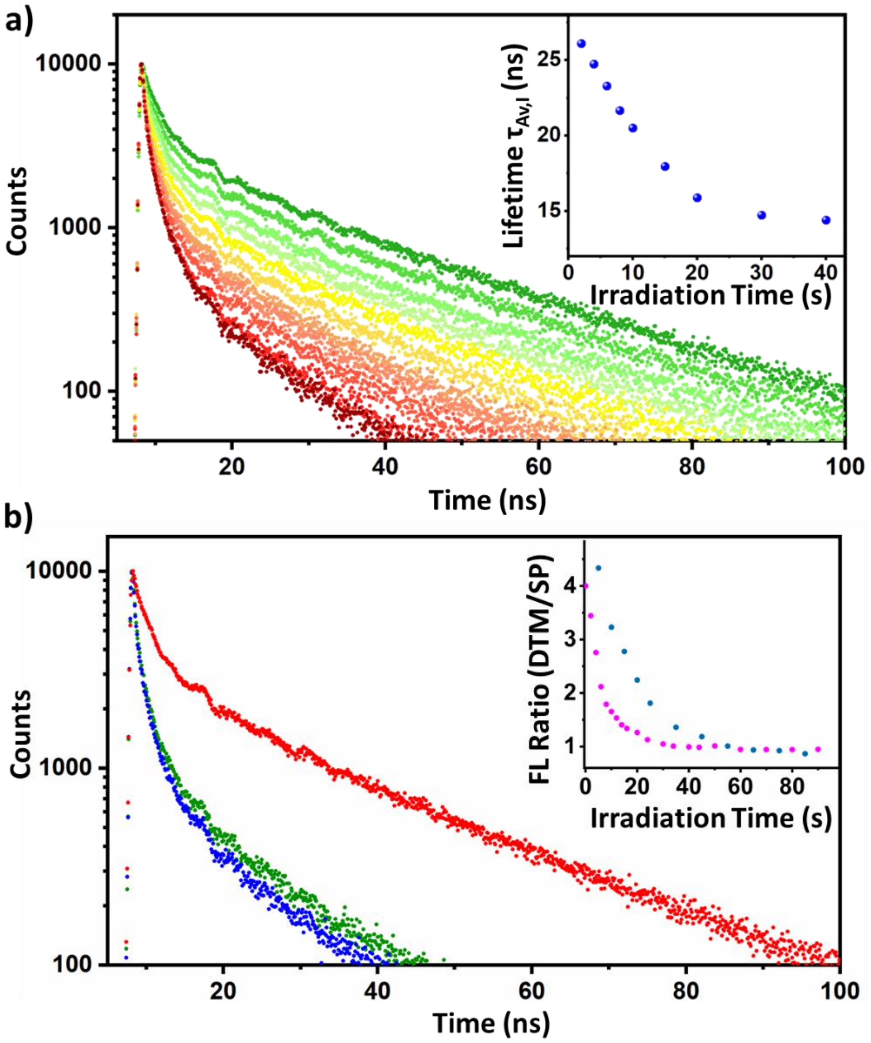

Supplementary Figure 14| a. Fluorescence lifetime decay of nanogel N4 after UV light irradiation for 0-100 s (C_{N4} = 0.5 mg mL^{-1}, λ_{ex}=375 nm, λ_{em} = 510 nm; Slit: Ex. = 1, Em. = 1); b. Fluorescence lifetime decay of N4 after irradiation with different UV lights for 120 s (C_{N4} = 0.5 mg mL^{-1}, λ_{ex} = 375 nm, λ_{em} = 510 nm; Slit: Ex. = 1, Em. = 1); UV lamp 1: 365 nm, 6 W; UV lamp 2 (LED): 365 nm, 4 W. Inserted plot: emission intensity ratios of DTM and SP channels in nanogel N4 during different UV irradiation for 0-100 s (C_{N4} = 0.5 mg mL^{-1}, $\lambda_{ex.}$ = 375 nm, slit width: Ex. = 1 nm, Em. = 1 nm).

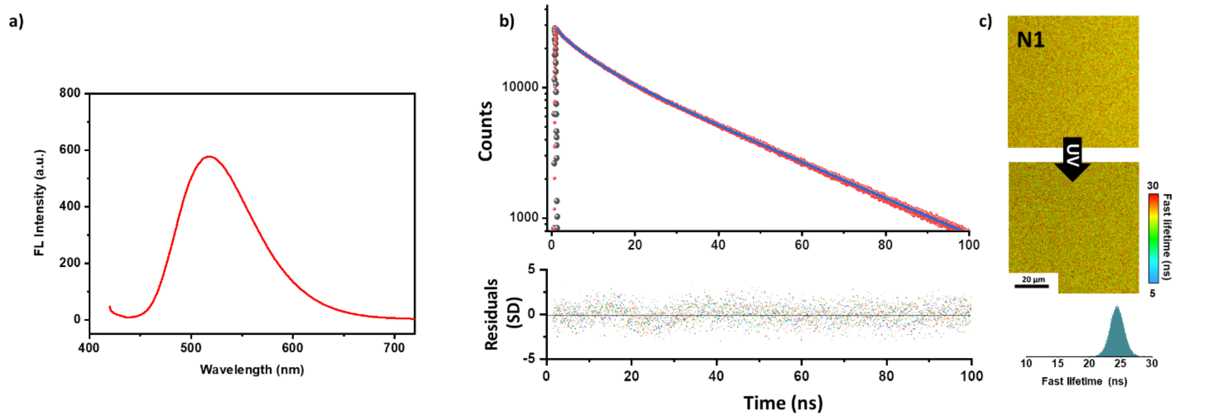

Supplementary Figure 15| a. Steady-state emission of nanogel N1 (λ_{ex} = 410 nm); b. Fluorescence lifetime decay spectra (points) with fitting (line) and residuals (bottom) for nanogel solution (N1); c) FLIM images and histogram of the nanogel solution before and after UV irradiation (λ_{ex} = 410 nm). The images were recorded three times and similar results were obtained.

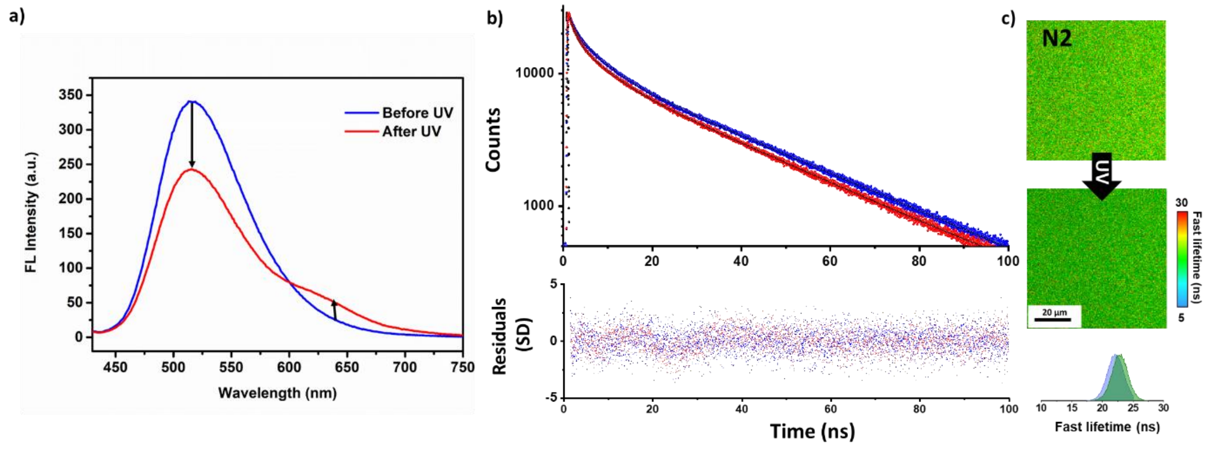

Supplementary Figure 16| a. Steady-state emission of nanogel N2 (λ_{ex} = 410 nm); b. Fluorescence lifetime decay spectra (points) with fitting (line) and residuals (bottom) for nanogel solution (N2); c) FLIM images and histogram of the nanogel solution before and after UV irradiation (λ_{ex} = 410 nm). The images were recorded three times and similar results were obtained.

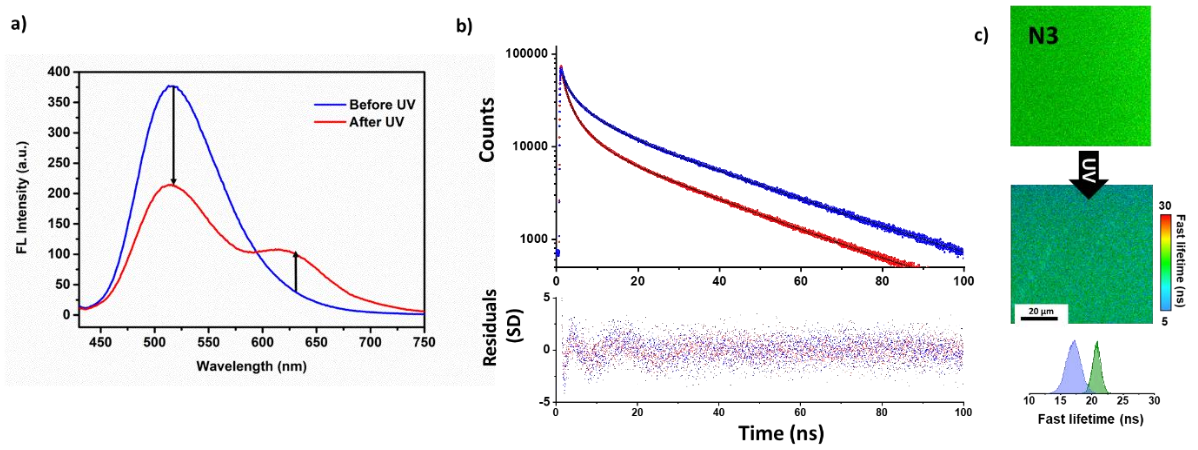

Supplementary Figure 17| a. Steady-state emission of nanogel N3 (λ_{ex} = 410 nm); b. Fluorescence lifetime decay spectra (points) with fitting (line) and residuals (bottom) for nanogel solution (N3); c. FLIM images and histogram of the nanogel solution before and after UV irradiation (λ_{ex} = 410 nm The images were recorded three times and similar results were obtained.

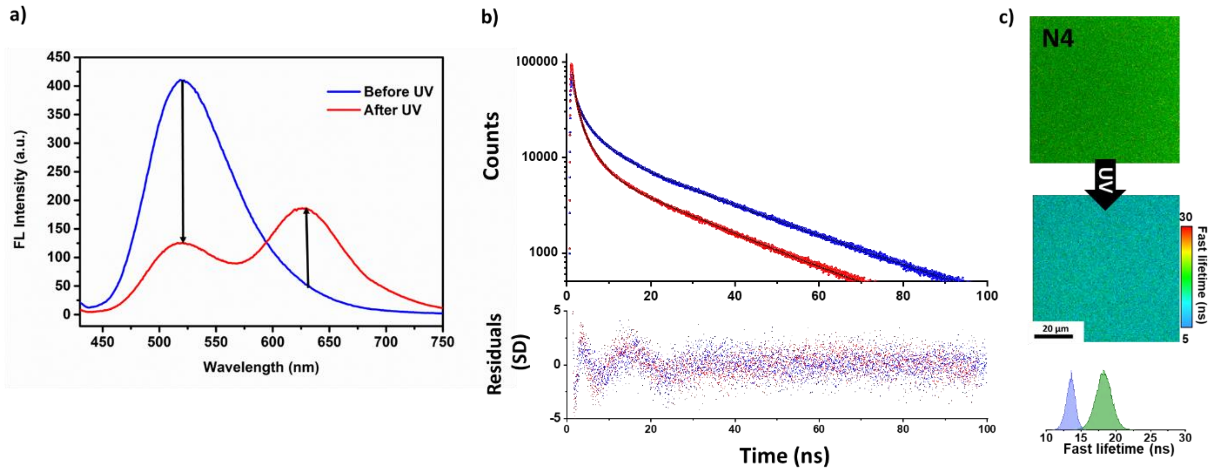

Supplementary Figure 18| a. Steady-state emission of nanogel N4 (λ_{ex} = 410 nm); b. Fluorescence lifetime decay spectra (points) with fitting (line) and residuals (bottom) for nanogel solution (N4); c. FLIM images and histogram of the nanogel solution before and after UV irradiation (λ_{ex} = 410 nm). The images were recorded three times and similar results were obtained.

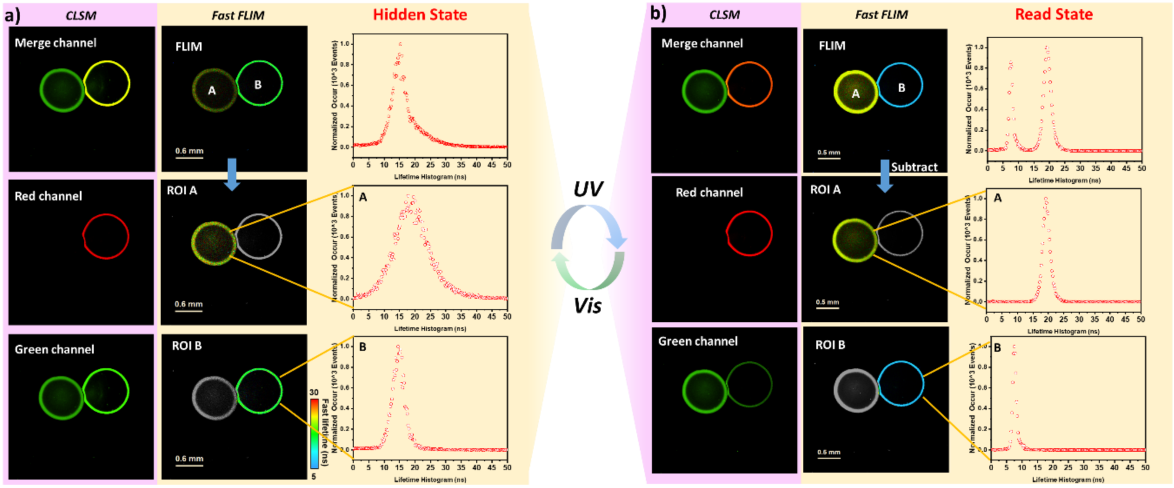

Supplementary Figure 19| FLIM and CLSM imaging used to identify the relative position and any nanogel information incorporated in the PVA film (solid state). Different areas of the nanogel were selected as regions of interest (ROI) and analysis of the corresponsive fluoresce lifetime for each ROI is shown. Left column: CLSM images of two nanogel droplet films with different channels (Green channel: λ_{ex} = 405 nm, λ_{em} = 480-550 nm; red channel: λ_{ex} = 405 nm, λ_{em}:600-700 nm); Right column: FLIM images of two nanogel droplet films with overall lifetime histogram and separated ROI with a subtracted lifetime histogram. (a) “Hidden” state (Before UV irradiation); (b) “Read” state (After UV irradiation).

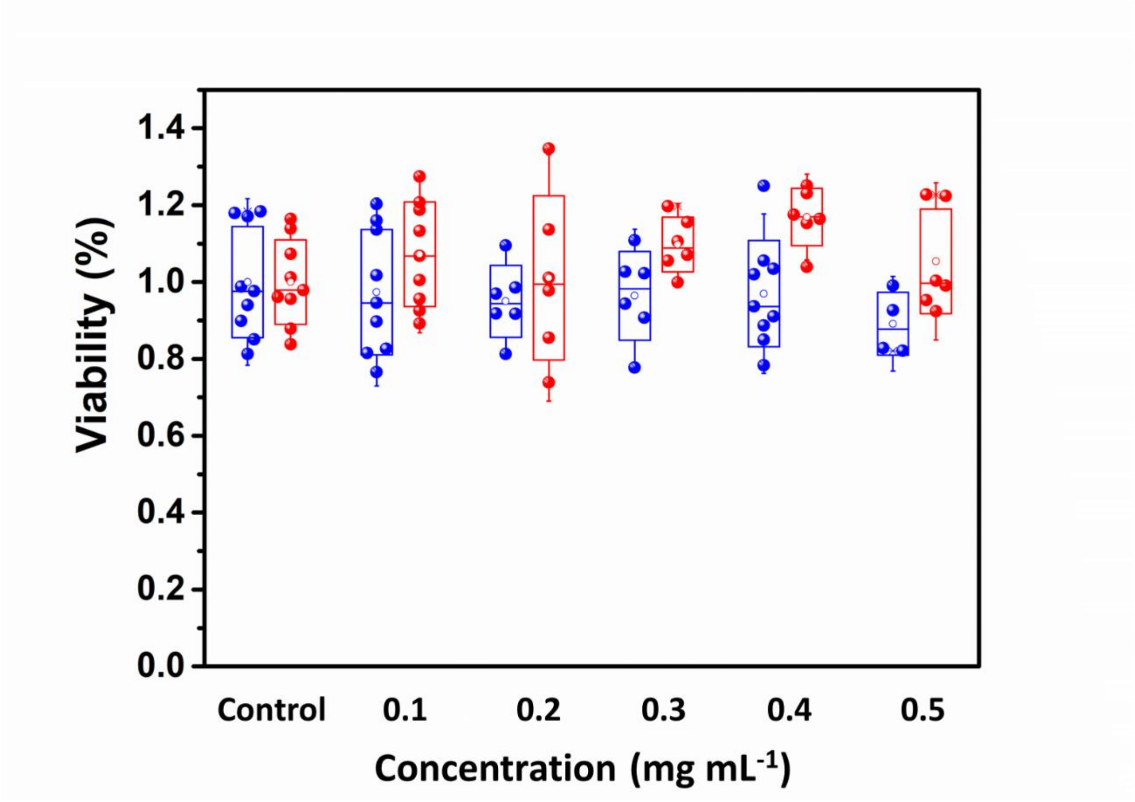

Supplementary Figure 20| Evaluation of the cytotoxicity of nanogels in A549 lung cancer cells. (Blue: nanogel N4, Red: nanogel without SPMA and DTMMA). Cell viability was assessed by 10% PrestoBlue viability assay following the supplier instructions after 24 h incubation with nanogel solution at 37 °C and 5% CO_2. The fluorescence intensity (FI) was detected in a FluoStar Omega microplate reader (BMG Labtech) (λ_{ex} = 530 nm, λ_{em} = 590 nm). Cell data are reported as viability % in comparison to the control sample. Nanogel only contained MMA and EGDMA was chosen as the control to explore the cytotoxicity of the SPMA and DTMMA. (Red: N6, blue: control). Data are presented as mean ± SD (N =3). Error bars represent standard deviation.

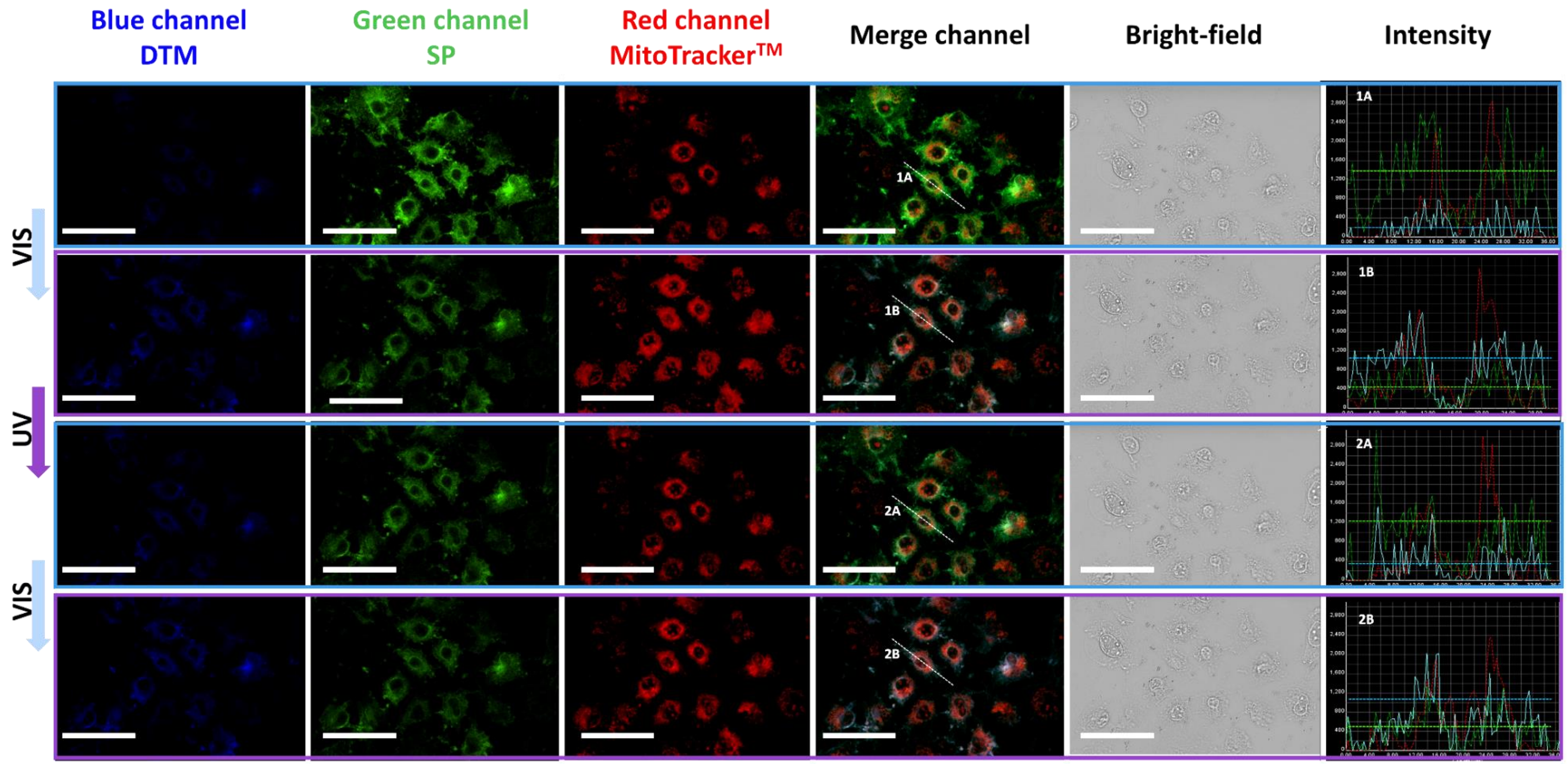

Supplementary Figure 21| Confocal fluorescence images of the non-specific accumulation of nanogel (N6) in the live A549 cells co-incubated with MitoTracker Red for 1 hour. The images between different rows were acquired by alternating the UV and vis irradiation for 120s. The intensity of region of interests (ROI) in different channels was plotted to quantify the emission change after light irradiations. (Blue channel: DTM, λ_{ex} = 405 nm; Green channel: Ring-opened form of SP, λ_{ex} = 405 nm; Red channel: MitoTracker Red, λ_{ex} = 630 nm; Scale bar = 40 µm).

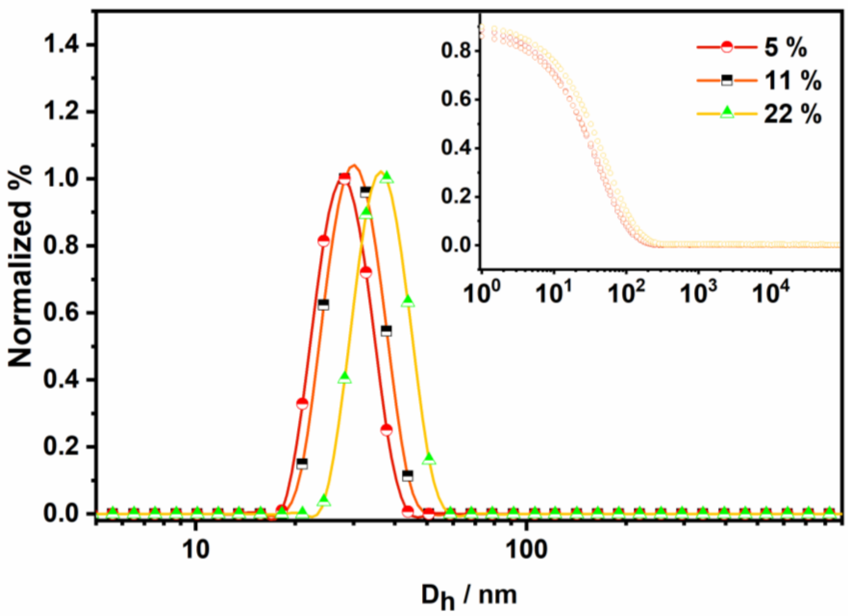

Supplementary Figure 22| Intensity-weighted size distribution and corresponding hydrodynamic diameters obtained of different ratio of azide functionality at 1 mg mL^{-1}, obtained by DLS (detection angle = 173°) and the corresponding autocorrelation function (inset). All determinations were repeated 4 times with 15 measurements recorded for each run. D_h values were calculated using the Stokes-Einstein equation where particles are assumed to be spherical.

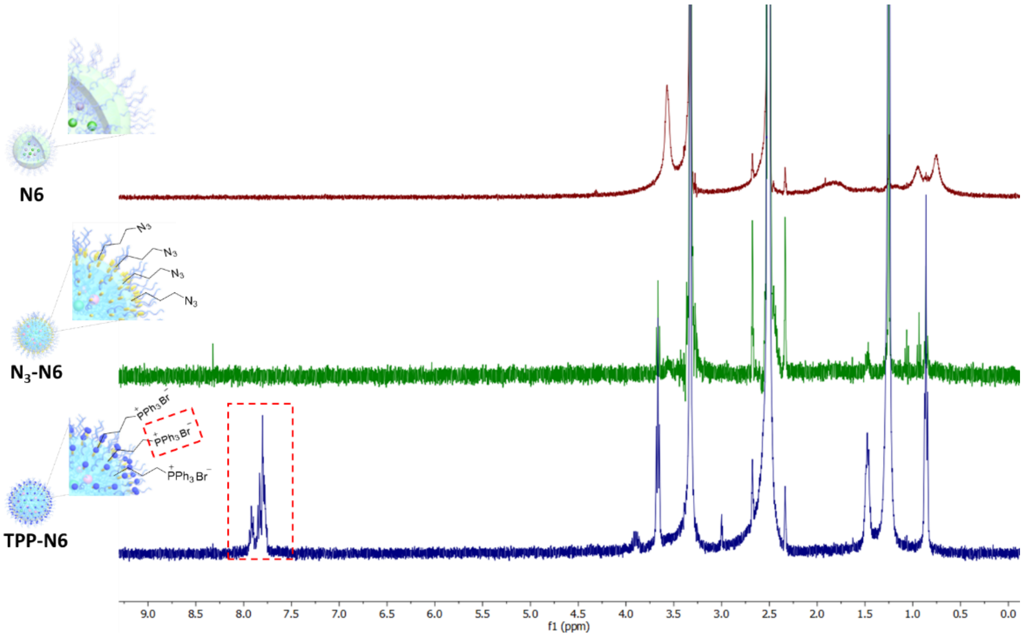

Supplementary Figure 23| Overlaid ^{1}H NMR spectra of nanogel with different functionality to confirm the TPP functionality. The new multiplet around 7.5-8.1 ppm of TPP functionalized nanogel corresponded to aromatic protons of triphenylphosphonium unit (DMSO-d_6, 400 MHz, 298K).

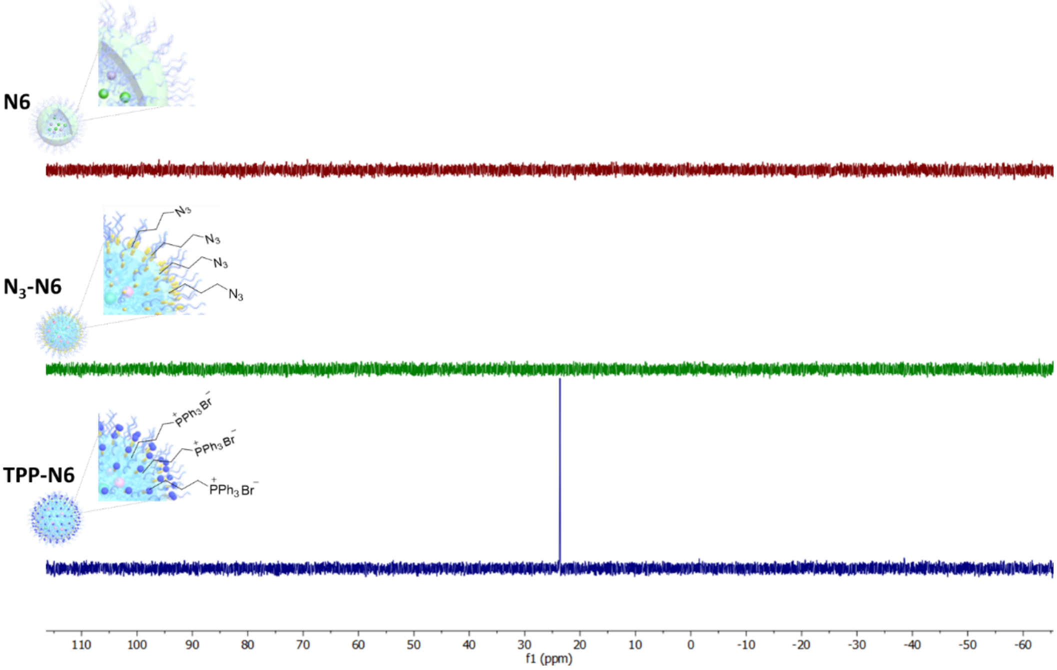

Supplementary Figure 24| ^{31}P NMR of nanogel with different functionality. The new peak in TPP functionalized nanogel corresponded to the triphenylphosphonium unit (DMSO-d_6, 400 MHz, 298K).

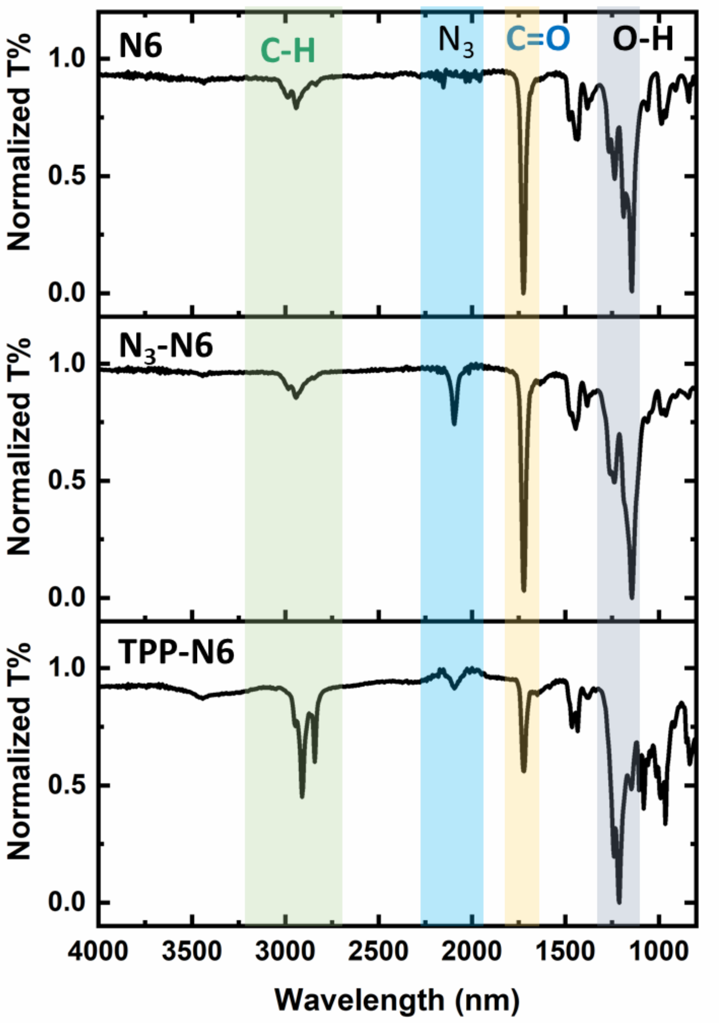

Supplementary Figure 25| The functionalization of nanogel azide and TPP was first assessed by FT-IR spectra. 16 Scans were recorded from 600 to 4000 cm^{-1} at a resolution of 4 cm^{-1}. In the azide-functionalized nanogel, the increase of the peak around 2100-2200 cm^{-1} corresponded to the stretching vibrations of azide group in N_3-N6 nanogel. This peak was further decreased after the click reaction with (But-3-yn-1-yl) triphenylphosphonium bromide which further confirmed the successful modification of TPP group.

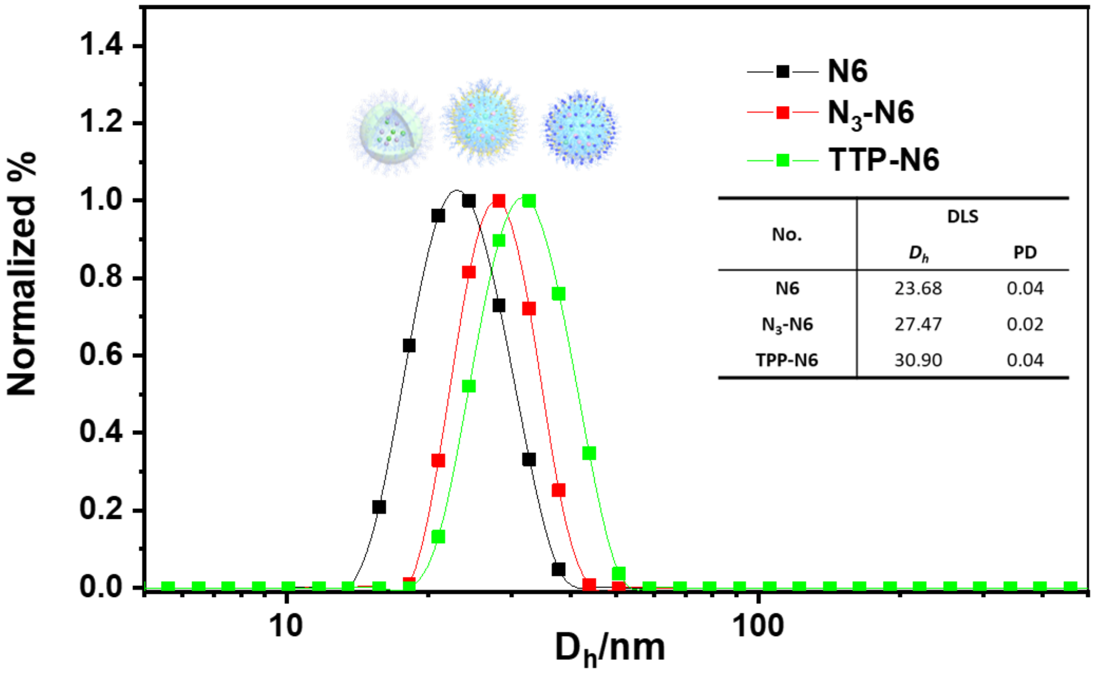

No.	DLS	
	D_h	PD
N6	23.68	0.04
N_3-N6	27.47	0.02
TPP-N6	30.90	0.04

Supplementary Figure 26| Intensity-weighted size distribution and corresponding hydrodynamic diameters obtained of unmodified nanogel (N6, black), azide functionalized nanogel (N_3-N6, red) and TPP functionalized nanogel (TPP-N6, green) at 1 mg mL^{-1}, obtained by DLS (detection angle = 173°). All determinations were repeated 4 times with 15 measurements recorded for each run. D_h values were calculated using the Stokes-Einstein equation where particles are assumed to be spherical.

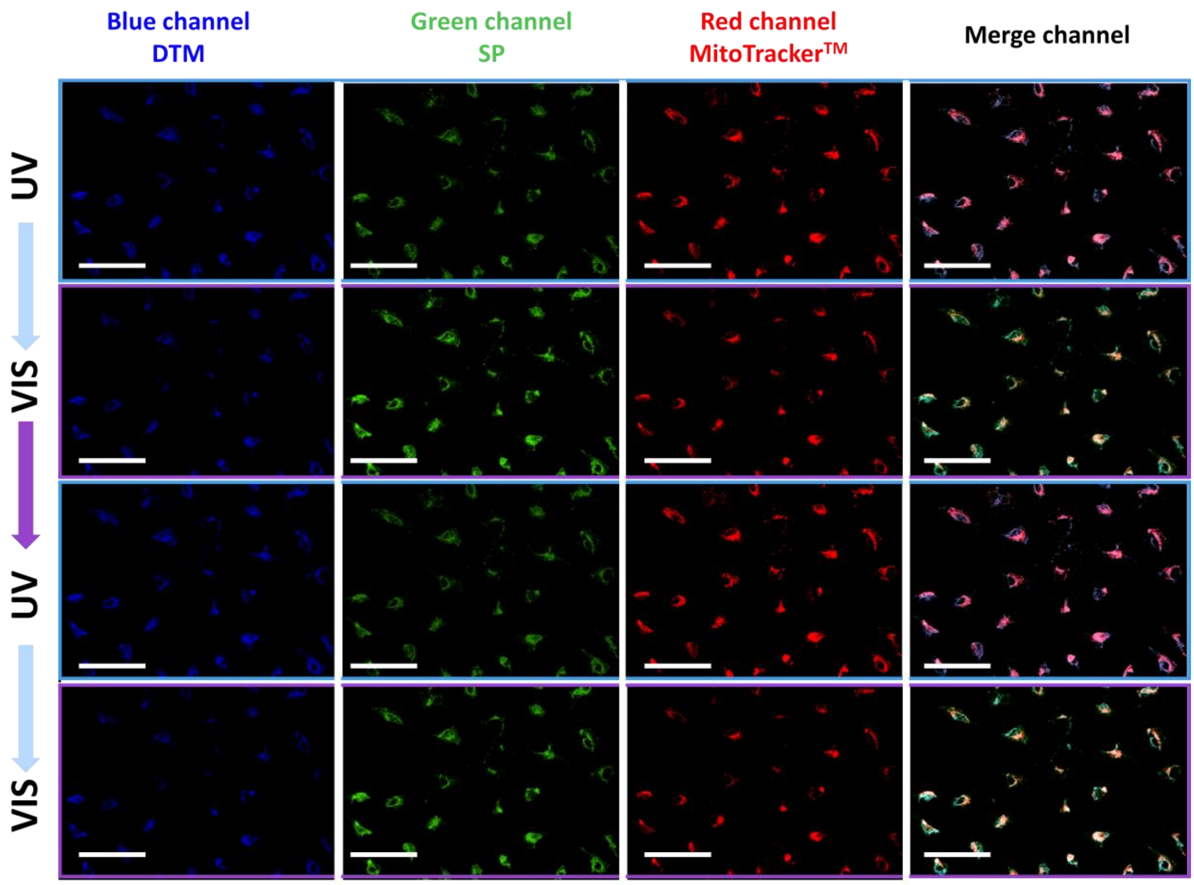

Supplementary Figure 27| Subcellular imaging of the nanogel (TPP-N6) in the live A549 cells co-incubated with MitoTracker Red using confocal microscopy. The intracellular images between different rows were acquired by alternating the UV and vis irradiation. (Blue channel: DTM, λ_{ex} = 405 nm; Green channel: Ring-opened form of SP, λ_{ex} = 405 nm; Red channel: MitoTracker Red, λ_{ex} = 630 nm; Scale bar = 60 μm).

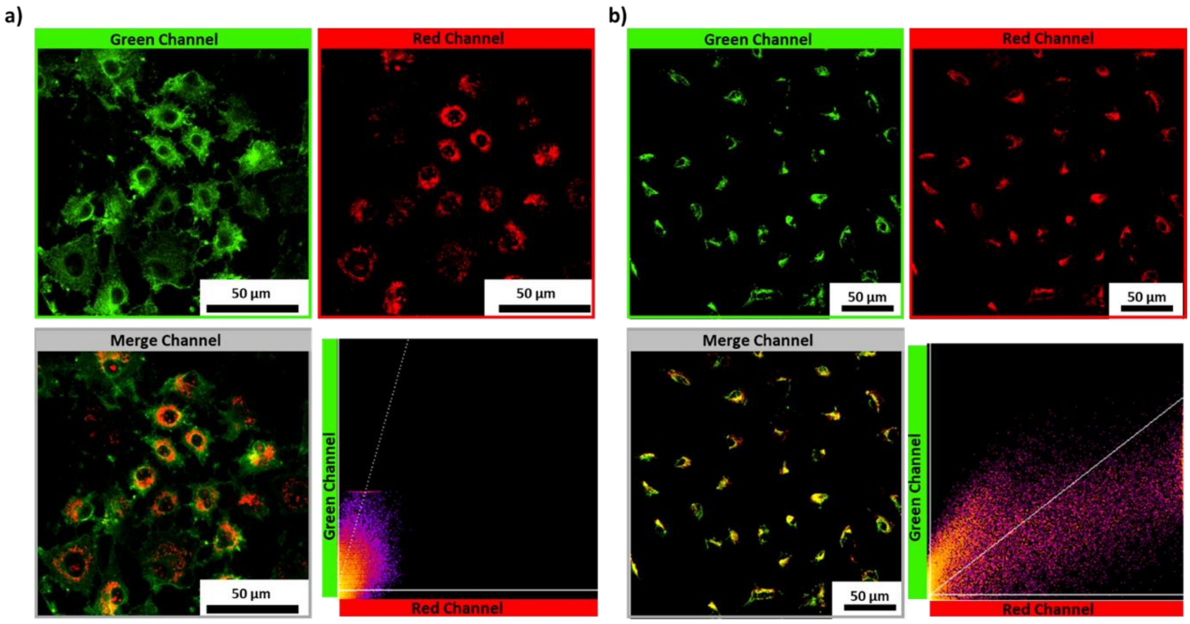

Supplementary Figure 28| a. Co-localization of nanogel (N6, green channel) with MitoTracker Red (Red channel) in live A549 cells; b. Co-localization of nanogel (TPP-N6, green channel) with MitoTracker Red (Red channel) in live A549 cells. Green channel: the ring-opened form of SP (λ_{ex} = 405 nm); Red channel: MitoTracker Red (λ_{ex} = 630 nm). The intensity correlation plots of TPP-N6 and MitoTracker Red was processed on imageJ and analyzed the Pearson Correlation Coefficient using Coloc2 plugins.

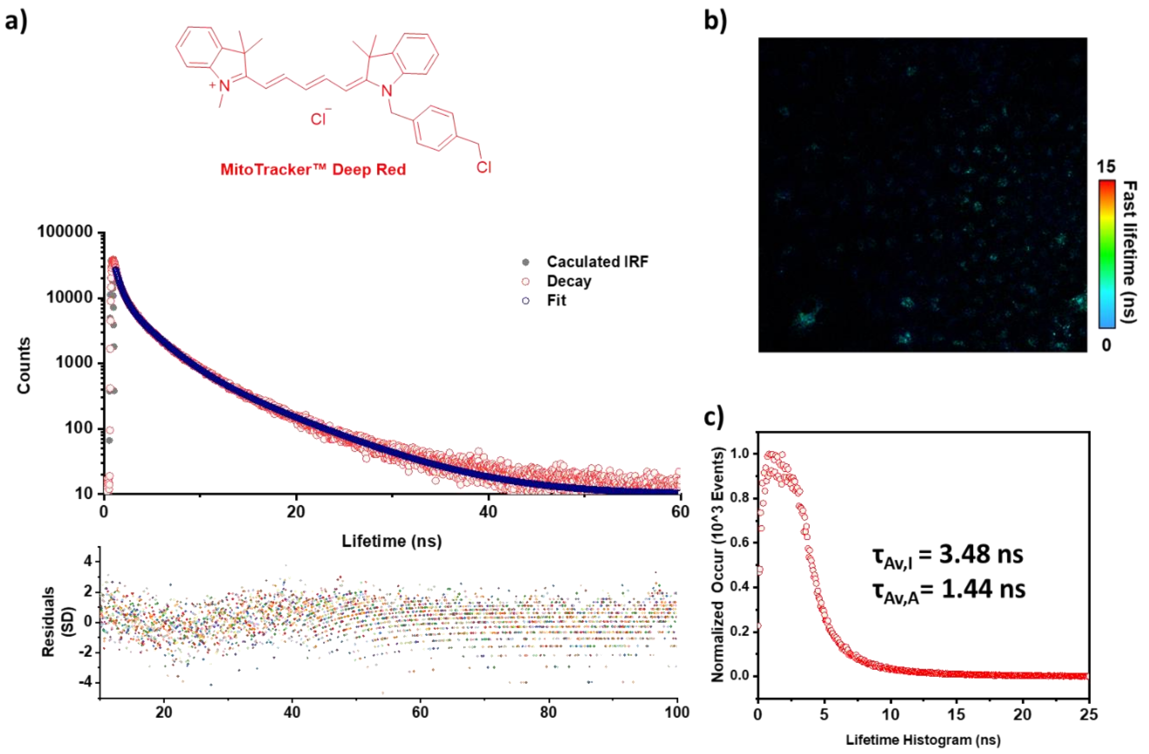

Supplementary Figure 29| a. Fluorescence lifetime decay (top) with fitting and residuals (bottom) of the Mito Tracker Cy5 in live A549 cells. b. FLIM imaging of the Mito Tracker Cy5 in live A549 cells.; c. Fluorescence lifetime histograms of the Mito Tracker Cy5 in the whole image range (λ_{ex} = 405 nm).

3,4-dibromo-1-(3-hydroxypropyl)-1H-pyrrole-2,5-dione (2).

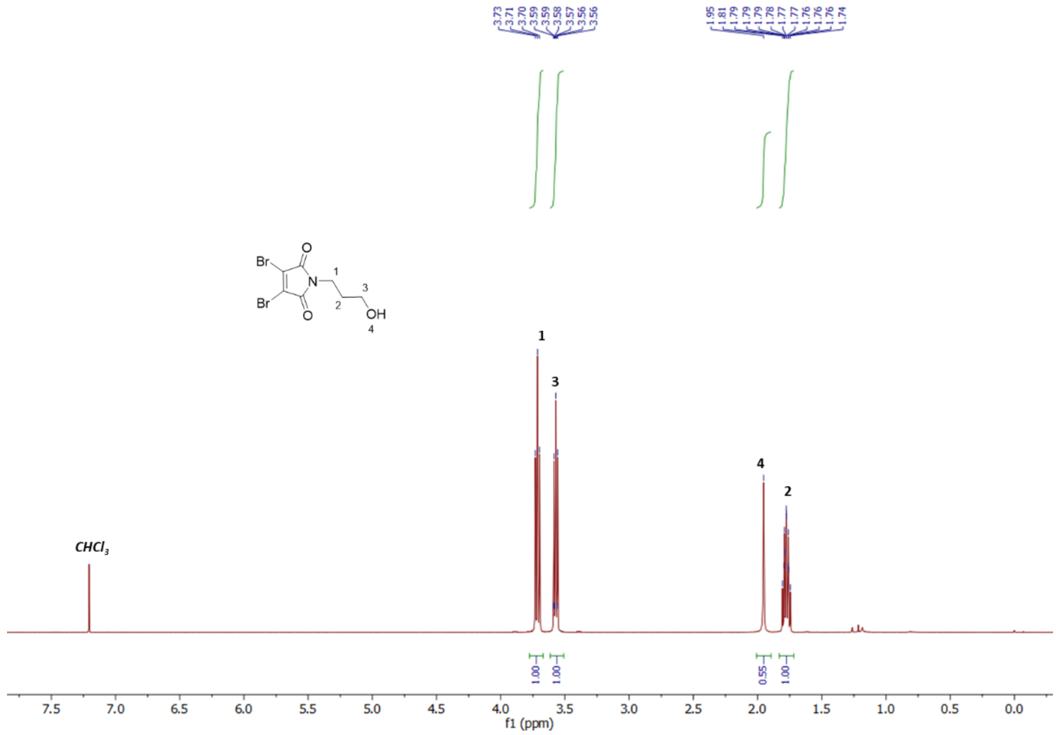

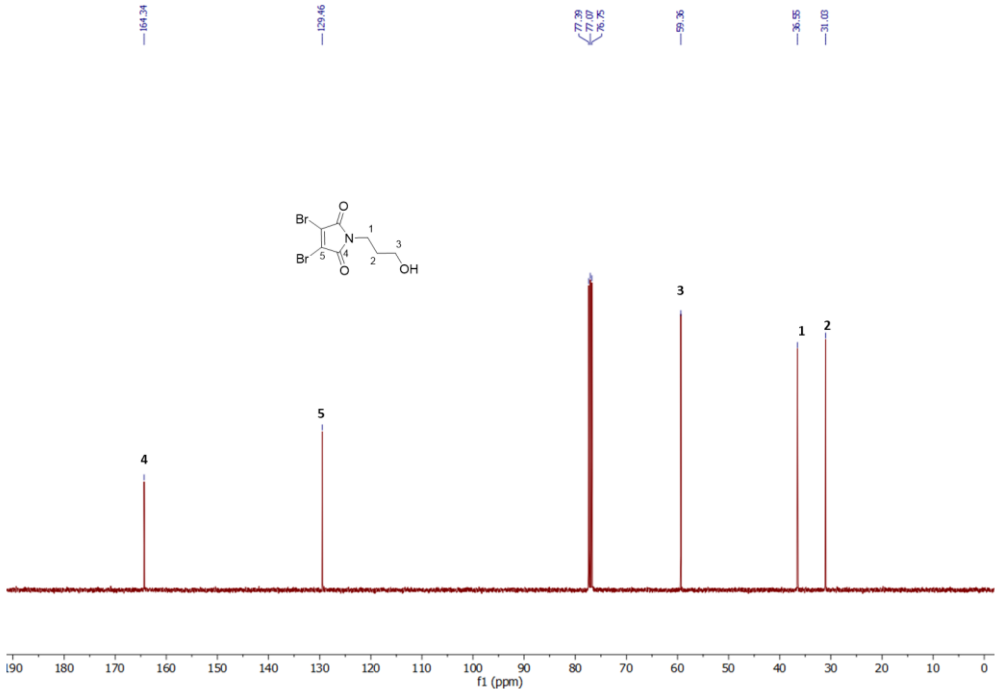

Supplementary Figure 30| ^{1}H NMR and ^{13}C NMR spectra of 3,4-dibromo-1-(3-hydroxypropyl)-1H-pyrrole-2,5-dione in $CDCl_3$ (400MHz, 298 K).

3,4-bis(butylthio)-1-(3-hydroxypropyl)-1H-pyrrole-2,5-dione (3)

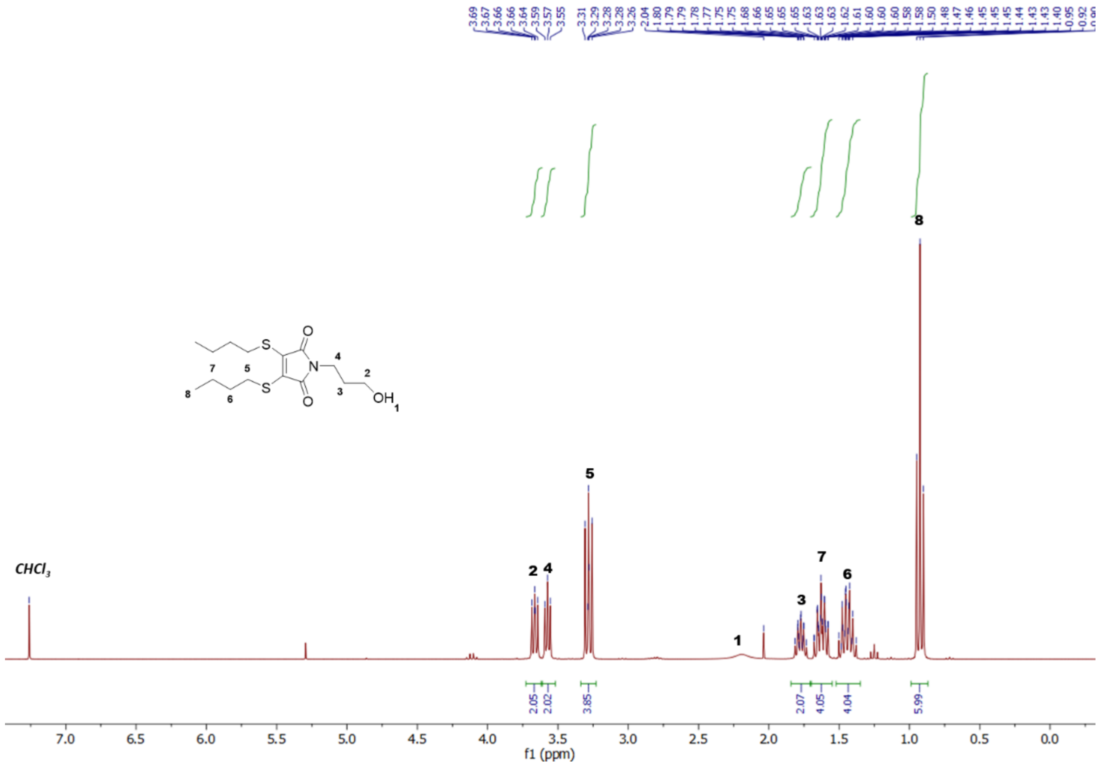

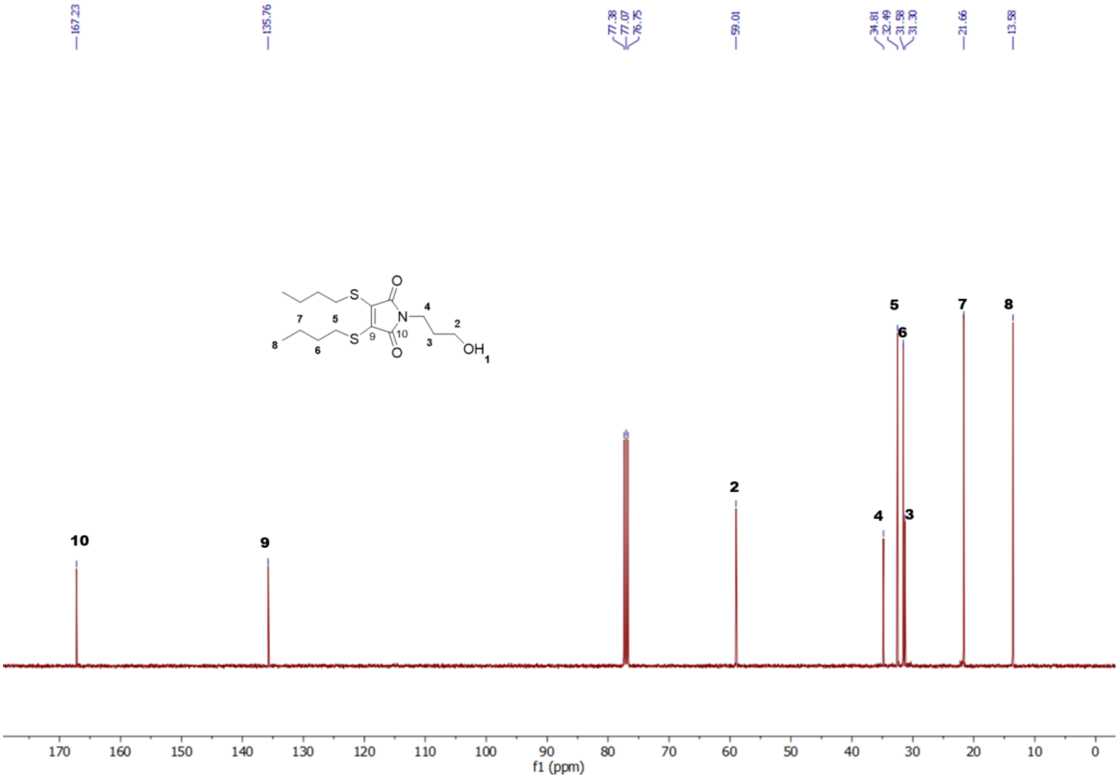

Supplementary Figure 31| ^{1}H NMR and ^{13}C NMR spectra of 3,4-bis(butylthio)-1-(3-hydroxypropyl)-1H-pyrrole-2,5-dione in $CDCl_3$ (400MHz, 298 K).

3-(3,4-bis(butylthio)-2,5-dioxo-2,5-dihydro-1H-pyrrol-1-yl)propyl methacrylate (DTMMA).

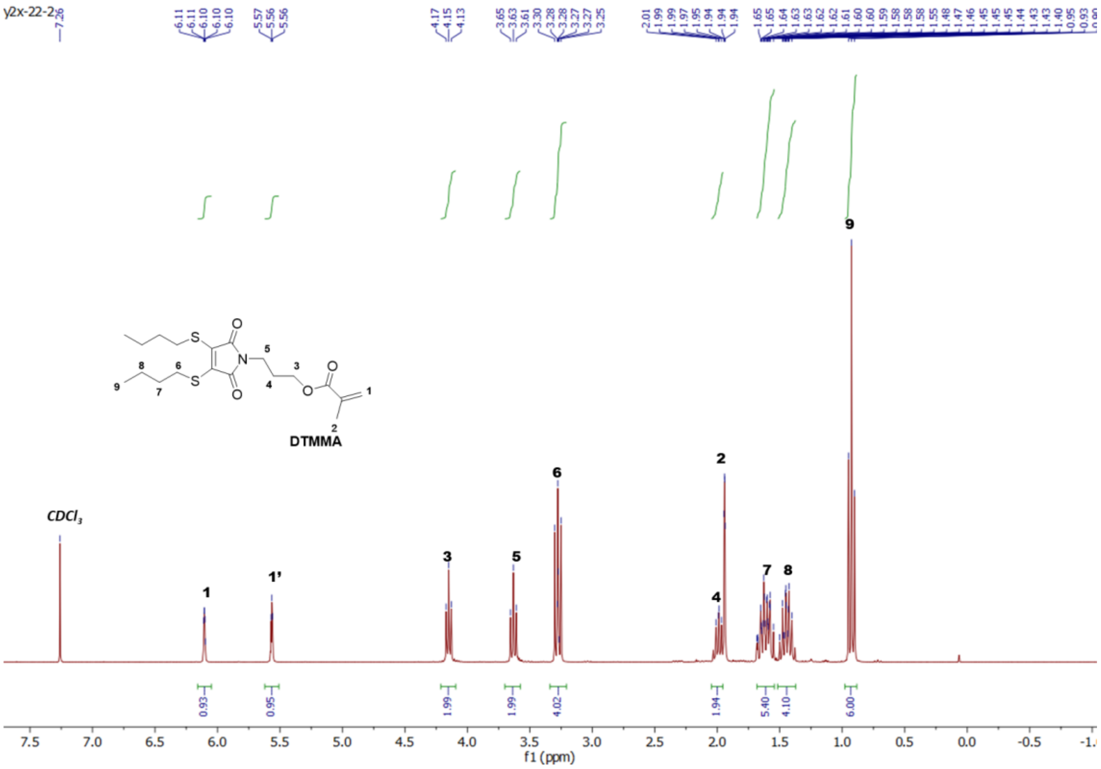

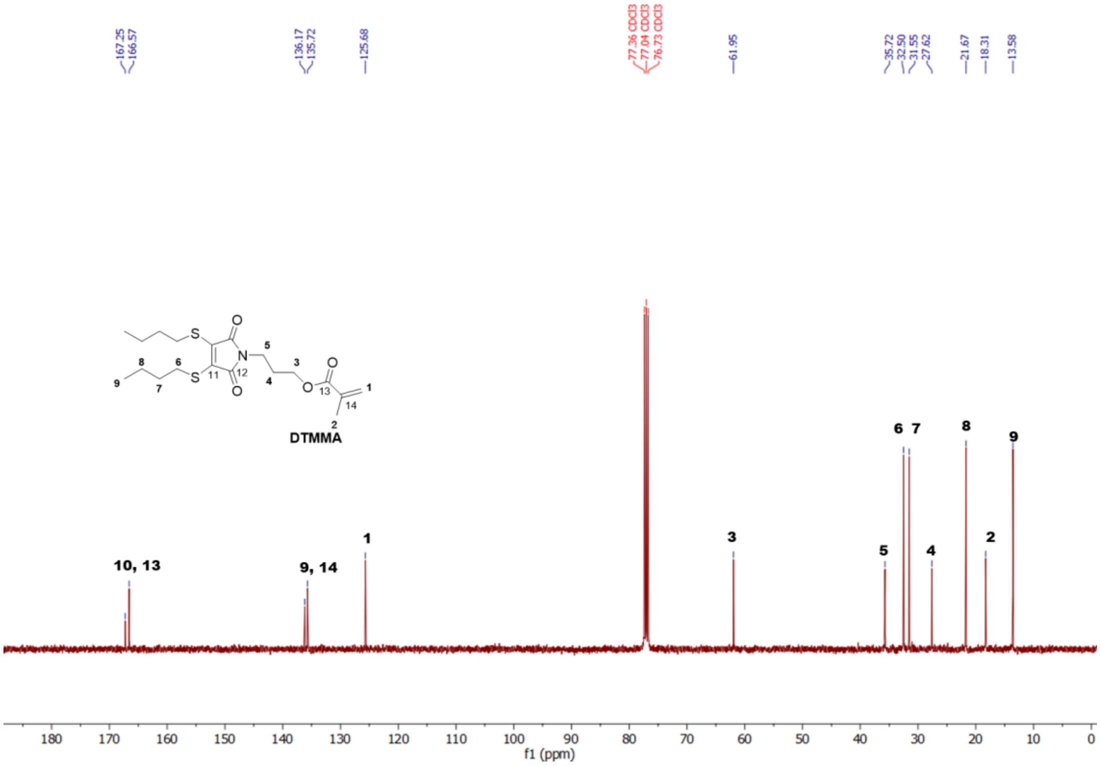

Supplementary Figure 32| ^{1}H NMR and ^{13}C NMR spectra of 3-(3,4-bis(butylthio)-2,5-dioxo-2,5-dihydro-1H-pyrrol-1-yl)propyl methacrylate in $CDCl_3$ (400MHz, 298 K).

9,9-dimethyl-2,3,9,9a-tetrahydrooxazolo[3,2-a]indole (5).

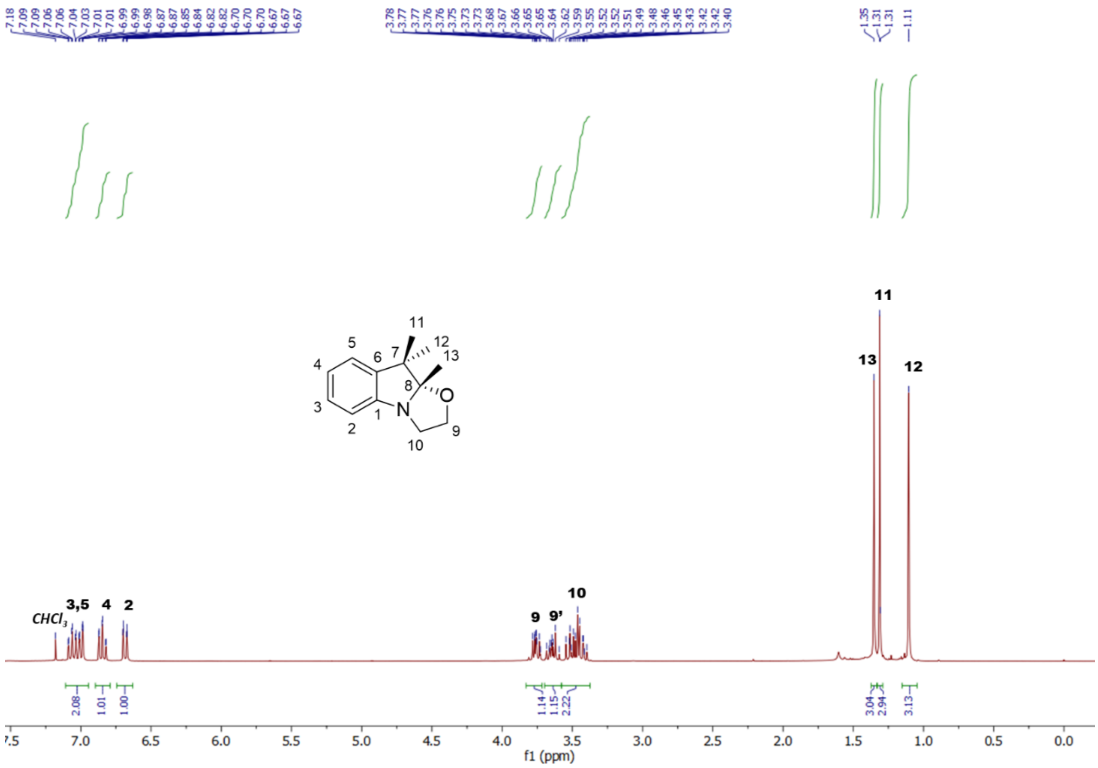

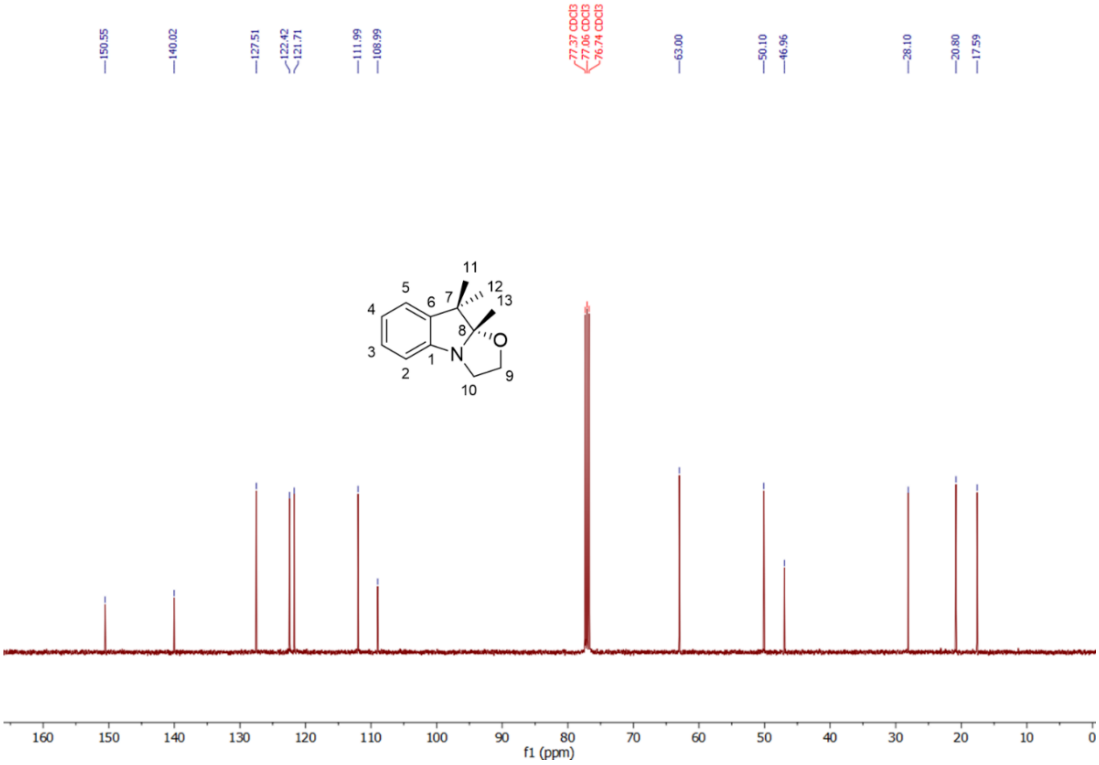

Supplementary Figure 33| ^{1}H NMR and ^{13}C NMR spectra of 9,9-dimethyl-2,3,9,9a-tetrahydrooxazolo[3,2-a]indole in $CDCl_3$ (400MHz, 298 K).

2-(3',3'-dimethyl-6-nitrospiro[chromene-2,2'-indolin]-1'-yl)ethan-1-ol (6)

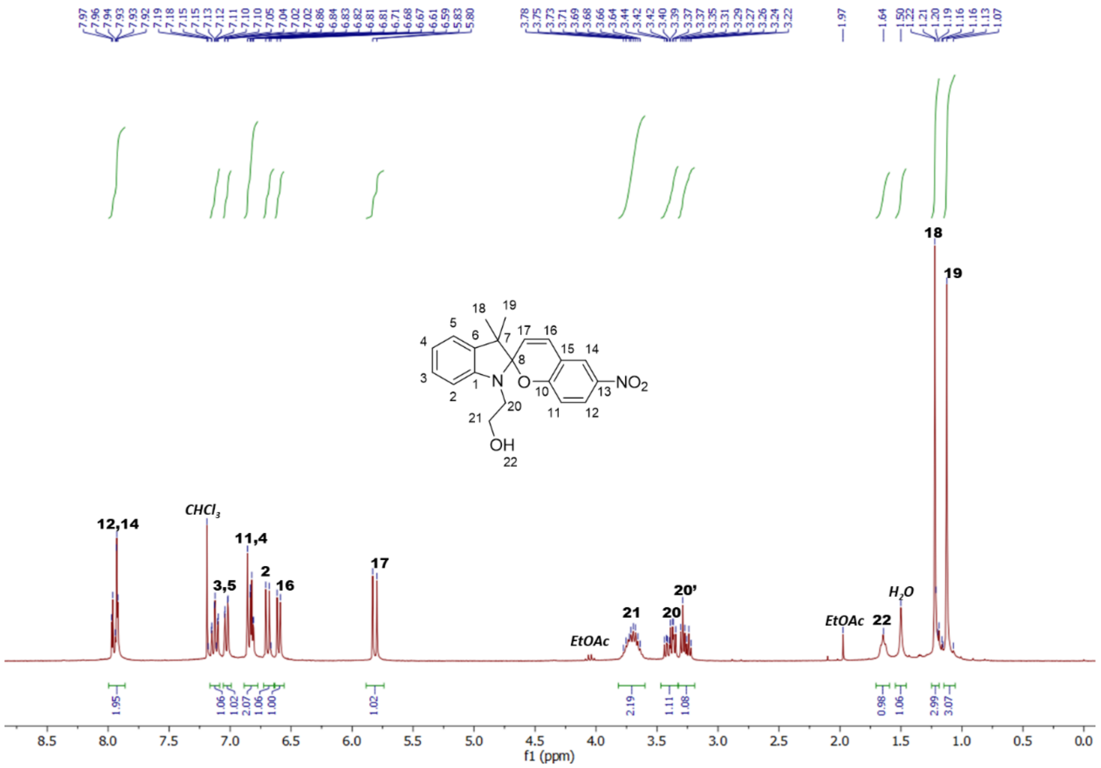

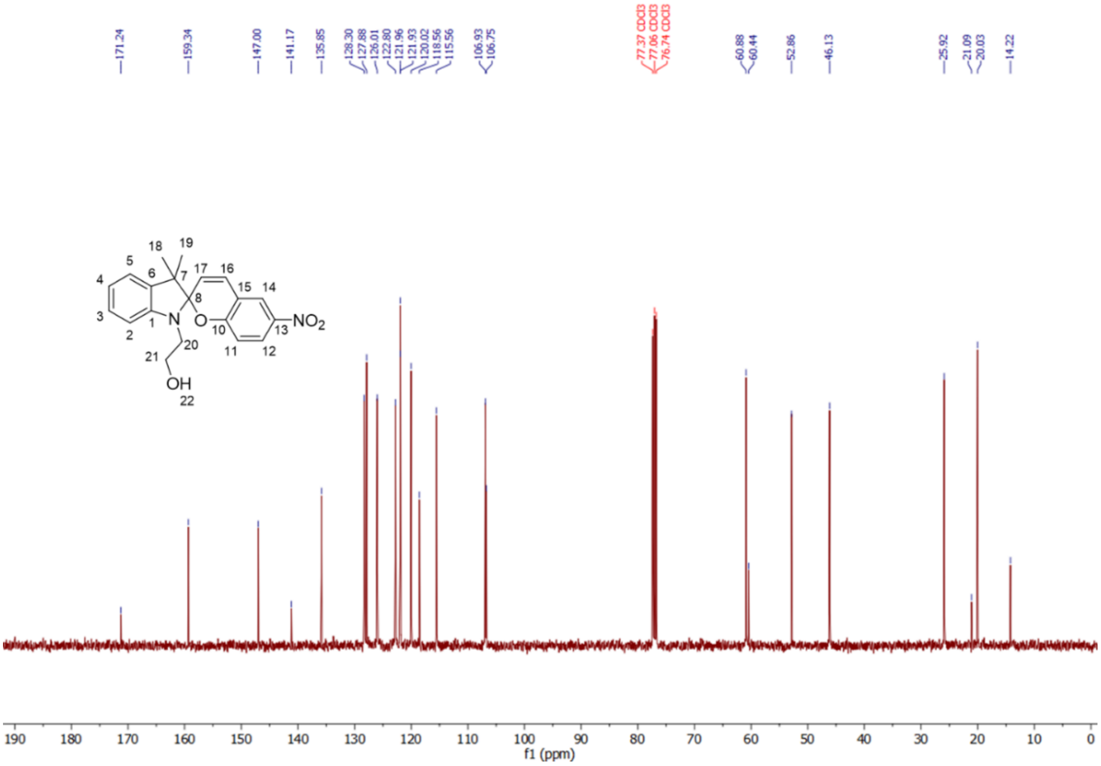

Supplementary Figure 34| ^{1}H NMR and ^{13}C NMR spectra of 2-(3',3'-dimethyl-6-nitrospiro[chromene-2,2'-indolin]-1'-yl)ethan-1-ol in $CDCl_3$ (400MHz, 298 K).

2-(3',3'-dimethyl-6-nitrospiro[chromene-2,2'-indolin]-1'-yl)ethyl methacrylate (SPMA)

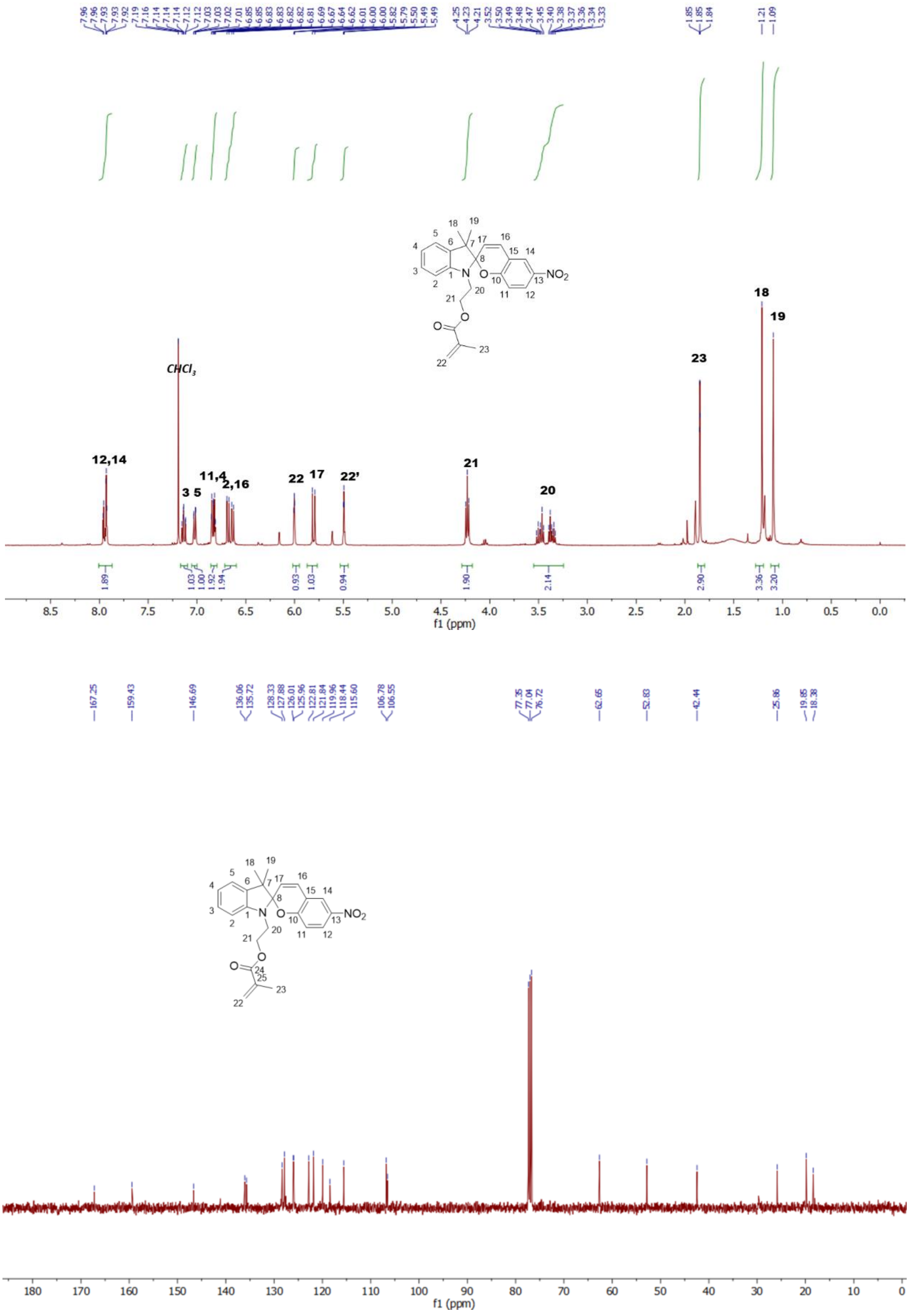

Supplementary Figure 35| ^{1}H NMR and ^{13}C NMR spectra of 2-(3',3'-dimethyl-6-nitrospiro[chromene-2,2'-indolin]-1'-yl)ethyl methacrylate in $CDCl_3$ (400MHz, 298 K).

Supplementary References

1. Robin MP*, et al.* New Functional Handle for Use as a Self-Reporting Contrast and Delivery Agent in Nanomedicine. *J Am Chem Soc* **135**, 9518-9524 (2013).

2. Beyer C, Wagenknecht HA. Synthesis of spiropyrans as building blocks for molecular switches and dyads. *J Org Chem* **75**, 2752-2755 (2010).

3. Sakata T, Yan Y, Marriott G. Family of Site-Selective Molecular Optical Switches. *J Org Chem* **70**, 2009-2013 (2005).

4. Jorgensen WL, Tiradorives J. The OPLS potential functions for proteins - energy minimizations for crytsals of cyclic-peptides and crambin. *J Am Chem Soc* **110**, 1657-1666 (1988).

5. Jorgensen WL, Maxwell DS, TiradoRives J. Development and testing of the OPLS all-atom force field on conformational energetics and properties of organic liquids. *J Am Chem Soc* **118**, 11225-11236 (1996).

6. Becke AD. Density-functional thermochemistry .3. the role of exact exchange. *J Chem Phys* **98**, 5648-5652 (1993).

7. Stephens PJ, Devlin FJ, Chabalowski CF, Frisch MJ. Ab-initio calculation of vibrational absorption and circular-dichroism spectra using density-functional force-fields. *J Phys Chem* **98**, 11623-11627 (1994).

8. Yanai T, Tew DP, Handy NC. A new hybrid exchange-correlation functional using the Coulomb-attenuating method (CAM-B3LYP). *Chem Phys Lett* **393**, 51-57 (2004).

9. Hehre WJ, Ditchfield R, Pople JA. Self-consistent molecular-orbital methods. 12. Further extensions of gaussian-type basis sets for use in molecular-orbital studies of organic-molecules. *J Chem Phys* **56**, 2257-2261 (1972).

10. Hehre WJ, Random L, Schleyer PvR, Pople JA. *Ab initio Molecular Orbital Theory*. Wiley (1986).

11. Zhao Y, Truhlar DG. The M06 suite of density functionals for main group thermochemistry, thermochemical kinetics, noncovalent interactions, excited states, and transition elements: two new functionals and systematic testing of four M06-class functionals and 12 other functionals. *Theor Chem Acc* **120**, 215-241 (2008).

12. Adamo C, Barone V. Toward reliable density functional methods without adjustable parameters: The PBE0 model. *J Chem Phys* **110**, 6158-6170 (1999).

13. Grimme S, Antony J, Ehrlich S, Krieg H. A consistent and accurate ab initio parametrization of density functional dispersion correction (DFT-D) for the 94 elements H-Pu. *J Chem Phys* **132**, 154104 (2010).

14. Grimme S, Ehrlich S, Goerigk L. Effect of the Damping Function in Dispersion Corrected Density Functional Theory. *J Comput Chem* **32**, 1456-1465 (2011).

15. Johnson ER, Becke AD. A post-Hartree-Fock model of intermolecular interactions: Inclusion of higher-order corrections. *J Chem Phys* **124**, (2006).

16. Cossi M, Rega N, Scalmani G, Barone V. Energies, structures, and electronic properties of molecules in solution with the C-PCM solvation model. *J Comput Chem* **24**, 669-681 (2003).

17. Tomasi J, Mennucci B, Cammi R. Quantum mechanical continuum solvation models. *Chem Rev* **105**, 2999-3093 (2005).

18. Adamo C, Jacquemin D. The calculations of excited-state properties with Time-Dependent Density Functional Theory. *Chem Soc Rev* **42**, 845-856 (2013).

19. Laurent AD, Adamo C, Jacquemin D. Dye chemistry with time-dependent density functional theory. *Phys Chem Chem Phys* **16**, 14334-14356 (2014).

20. Macromodel, Schödinger, LLC, New York, NY.) (2019).

21. Maestro, Schödinger, LLC, New York, NY.) (2019).

22. Frisch MJ*, et al.* Gaussian 16 Rev. C.01.) (2016).

23. Meer BWvd. Förster Theory. In: *FRET – Förster Resonance Energy Transfer* (ed Hildebrandt IMN) (2013).

24. Sun MJ*, et al.* In Situ Visualization of Assembly and Photonic Signal Processing in a Triplet Light-Harvesting Nanosystem. *J Am Chem Soc* **140**, 4269-4278 (2018).

25. Robin MP, Raymond JE, O'Reilly RK. One-pot synthesis of super-bright fluorescent nanogel contrast agents containing a dithiomaleimide fluorophore. *Mater Horiz* **2**, 54-59 (2015).
